# Identification of Melanoma Subsets Based on DNA Methylation Sites and Construction of a Prognosis Evaluation Model

**DOI:** 10.1155/2022/6608650

**Published:** 2022-10-11

**Authors:** Li Tengda, Qian Cheng, Sun Yi

**Affiliations:** ^1^School of Laboratory Medicine and Life Science, Wenzhou Medical University, Wenzhou 325035, Zhejiang, China; ^2^Key Laboratory of Laboratory Medicine, Ministry of Education, Wenzhou Medical University, Wenzhou 325035, Zhejiang, China; ^3^Zhejiang Provincial Key Laboratory for Medical Genetics, School of Laboratory Medicine and Life Science, Wenzhou Medical College, Wenzhou 325035, China; ^4^Department of Laboratory Medicine, Shanghai Municipal Hospital of Traditional Chinese Medicine, Shanghai University of Traditional Chinese Medicine, Shanghai 201203, China; ^5^Department of Laboratory Medicine, Shanghai General Hospital, Shanghai Jiao Tong University School of Medicine, Shanghai 200080, China

## Abstract

**Background:**

Melanoma is a lethal skin malignant tumor, and its formation or development is regulated by various genetic and epigenetic molecules. Although there are traditional methods provided for the doctors to evaluate the patients' prognosis or make the diagnosis, the novel method based on epigenetic markers is still needed to make the early diagnosis.

**Results:**

We identified 256 melanoma-independent prognosis-related methylation sites (*P* < 0.0001) and divided patients into seven methylation subgroups. Methylation levels and survival time in the C2 subgroup were lower than that of other clusters (*P* < 0.05). We established the predicted model of prognosis risk for melanoma using the significantly changed methylation sites in C2. The model efficiently divided patients into high- and low-risk groups (area under the receiver operating characteristic curve, 0.833). Risk scores and patient survival time were negatively correlated (*r*_*s*_ = −0.325, *P* < 0.0001). Genes corresponding to the independent prognosis-associated methylation sites were enriched in cancer- and immunology-related pathways. We identified 35 hub genes. *DOK2*, *GBP4*, *PSMB9*, and *NLRC5* were significantly changed according to methylation subgroups, survival, tumor stages, and T categories and were positively correlated, which was validated in the testing group (*P* < 0.05). The levels of *DOK2*, *GBP4*, *PSMB9*, and *NLRC5* had an opposite trend to their methylation sites in patients with poor prognosis.

**Conclusions:**

We identified seven DNA methylation subtypes and constructed a highly effective prognosis risk assessment model. The transcript levels of key genes corresponding to the independent prognosis-related methylation sites were significantly changed in patients according to prognosis and positively correlated with each other, indicating they may collaboratively promote melanoma formation. These findings further our understanding of the mechanism of melanoma and provide new targets for diagnosis and treatment.

## 1. Background

Melanoma is the most aggressive skin cancer and originates from malignant melanocytes [1]. Once melanoma has metastasized, it is generally associated with a poor prognosis [2]. The classification of melanoma has traditionally been based on histological features, and this approach divides cases into superficial spreading melanoma, lentigo maligna melanoma, nodular melanoma, and other variants [3–5]. Recently, new classification systems, such as TNM (primary tumor, lymph node involvement, distant metastases), have been developed [5–7]. Although these systems provide general information for clinicians to predict the prognosis of patients with melanoma and generate a reference for doctors to give appropriate treatment advice, early diagnosis of melanoma is still difficult. Furthermore, an accurate or consistent model to predict patient prognosis and identify personalized treatment strategies remains lacking [2, 4, 6–8].

DNA methylation is a common epigenetic change involved in multiple cellular processes. Three DNA methyltransferases (DNMTs) have been identified in advanced eukaryotes, and several studies have linked aberrant protein structures of DNMTs with abnormal embryonic development and cancer development [9, 10]. Previous research has shown that cancer-related DNA methylation events occur on CpG (5′-cytosine-phosphate-guanine-3′) islands and in 70% of mammalian promoter regions [9]. Methylation of CpG islands plays an important role in the regulation of gene transcription and is a critical factor of cellular malignant transformation [10, 11]. CpG hypermethylation in the promoter region can influence the transcription of genes. Many actively transcribed genes show high DNA methylation levels, indicating the background or spatial distribution of DNA methylation was important for the regulation of gene transcription and the formation of malignant disease [11].

Cancer genomics studies identified a recurrent mutation in DNMT3a in 25% of patients with acute myeloid leukemia [12] that affects the prognosis of patients [13]. These mutations were heterozygous and interfere with the catalytic activity of the enzyme. At present, the hypomethylating agent 5-azacytidine (azacytidine) shows good curative effect in myelodysplastic syndrome [9, 14]. In addition, one study showed that a prediction model for colon adenocarcinoma patient prognosis built by the DNA methylation sites identified patients with poor prognosis [15]. Few studies have examined DNA methylation in melanoma.

In this study, the methylation levels were examined in samples from 475 melanoma patients and the patients were divided into different methylation subtypes. A prognostic model was built based on the prognosis-related DNA methylation sites. These results may help provide a new method to assess the prognosis of patients and lay a theoretical foundation for researchers to understand this disease from a uniquely epigenetic perspective.

## 2. Methods

### 2.1. Download and Preprocessing of Data

**Figure 1:**
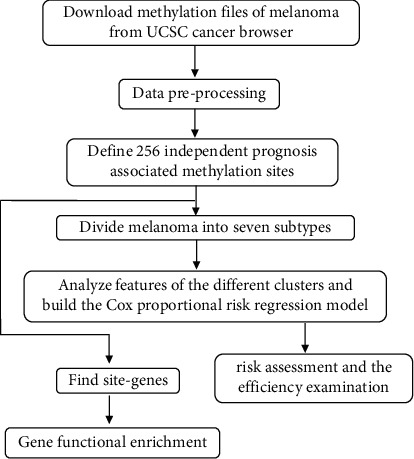
Flow chart of the study. UCSC, University of California Santa Cruz.

The study flow chart is shown in Figure 1. We downloaded transcriptome files of 471 patients with melanoma from The Cancer Genome Atlas (TCGA) database (https://portal.gdc.cancer.gov/) on May 7, 2021. The platform of transcriptome file was Illumina. Detailed information of patients is shown in Table 1. The detection platform for the 475 methylation data files was the Illumina Human Methylation 450; we acquired data from the University of California Santa Cruz (UCSC) cancer browser (https://xena.ucsc.edu/) on May 8, 2021. The testing data were from GSE98394. The exclusion criteria for the DNA methylation sites were as follows: there were more than 70% missing data in the whole sample; the sites located on sex chromosome; single nucleotide polymorphisms; the sites were not on the gene promoter region (2 kb upstream to 0.5 kb downstream of the transcription start site); and they were cross-reactive genome sites [16]. Clinical samples were excluded based on the following exclusion criteria: less than 30 days of follow-up data or no recorded follow-up data; no survival status; and critical clinical information such as tumor stage was missing or unknown. We used the R package impute and sva to perform the batch correction [17–19].

### 2.2. Division of DNA Methylation Subtypes of Melanoma

The univariate Cox proportional risk regression model was built by DNA methylation sites, patients' age, stage, gender, TNM classifications, grade, and the follow-up data; after calculating, we got the prognosis-related sites (Table S1). Those sites were used in the multivariate Cox regression models to get the independent prognosis-related methylation sites (Table S2), and they were analyzed by the ConsensusClusterPlus package [20] in R software to determine the melanoma subtypes. Based on the k-means, we divided each sample into k groups, and the repeated times was used to check the classifications' stability. Pairwise consensus values were calculated and recorded for each k value. We used the Euclidean squared distance metric to calculate the k-means, and the results matrix included over 100 iterations. The k value was determined if there were high consistency and low variation in the cluster matrix. Pheatmap package [21] was used to draw the heatmap. If the squares were diagonally distributed, the matrix consensus was perfect.

### 2.3. Construction of the Prognosis Prediction Model and Evaluation

We selected the subcluster with dramatically high or low survival probability and significantly changed methylation levels compared with other subclusters; if there were more sites in the targeted subcluster, those sites would be chosen to build the Cox proportional hazard model by coxph function in R software [22, 23].

### 2.4. Analysis of Gene Pathway Enrichment and Hub Genes

We used CytoHubba to predict or discover important genes. We drew the interaction map of genes using the String tool (https://string-db.org) and imported it into CytoHubba to calculate the scores of the total genes. The main parameters in this research were maximal clique centrality (MCC), depth, edge percolated component (EPC), and maximum neighborhood component (MNC). We selected the top 50 genes in each method and constructed a Venn diagram. The overlapping genes were identified as key genes and used for subsequent analysis.

### 2.5. Statistical Analyses

Comparison of the continuous data in three or more groups was performed by the Kruskal–Wallis test or analysis of variance according to whether it met normal distribution and homogeneity of variance. The comparison of numerical data in different groups was analyzed by the Chi-test. Pearson's or Spearman's correlation analysis was used to calculate the coefficients of two measurement data. Comparison of the levels of the methylation sites in different melanoma subtypes was performed by the Wilcoxon test. The survival analysis was performed by Survival package [24, 25] in R software. All statistical analyses were performed using IBM SPSS statistics 21.0 or the R software, and *P* < 0.05 was defined as statistically significant.

Other methods in this research were performed as the same as our published article [26].

## 3. Results

### 3.1. Clinical Characteristics of Patients with Melanoma and Filtering of Independent Prognostic Methylation Sites

This study included 290 male patients and 180 female patients (Table 1). The mean patient age was 58.2 years (with 250 cases ≤60 years old and 212 cases >60 years old, Table 1). The survival time was less than 1 year for 63 cases, 1 to 5 years for 246 cases, and more than 5 years in 151 cases (Table 1). Regarding TNM staging system, there were 23 cases in T0 stage, 42 cases in T1 stage, 78 cases in T2, 90 cases in T3 stage, and 153 cases in T4 stage (Table 1). Regarding distant metastasis, there were 418 patients in M0 stage and 24 patients in M1 stage; in terms of lymph node involvement, 235 patients were in N0 stage, 74 patients were in N1 stage, 49 patients were in N2 stage, and 55 patients were in N3 stage (Table 1). Among the 470 total patients, 77 cases were in stage I, 140 cases were in stage II, 171 cases were in stage III, and 23 cases were in stage IV (Table 1).

Combined with the clinical data, we performed Cox univariate regression analysis on selected 206,635 methylation sites in the melanoma samples and extracted 783 prognostic-related methylation sites (*P* < 0.0001, Table S1), and those sites were not independently associated with patients' prognosis. We then performed multivariate Cox regression analysis on the above 783 sites to identify the independent prognostic methylation sites and obtained 256 independent prognostic methylation sites (*P* < 0.0001, Table S2). Levels of the 256 sites and the follow-up were merged in Table S3. These sites were used for the construction of the subsequent risk assessment model and further analysis.

### 3.2. DNA Methylation Subtypes of Patients with Melanoma and the Clinical Features

**Figure 2:**
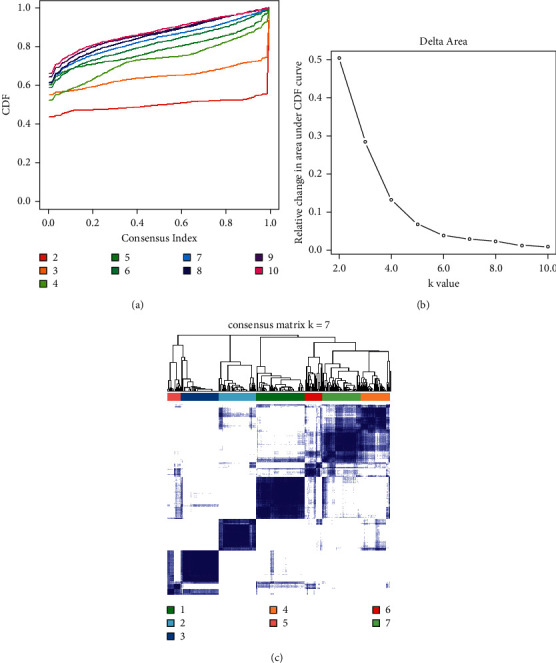
Conditions for determining the number of melanoma methylation subtypes. (a) CDF-consensus index graph. (b) Delta area chart. (c) The consistency matrix of subclusters when *k* = 7. In the figure, 1–7 represents subclusters from 1 to 7. CDF, consensus cumulative distribution function; *k*, the number of clusters.

**Figure 3:**
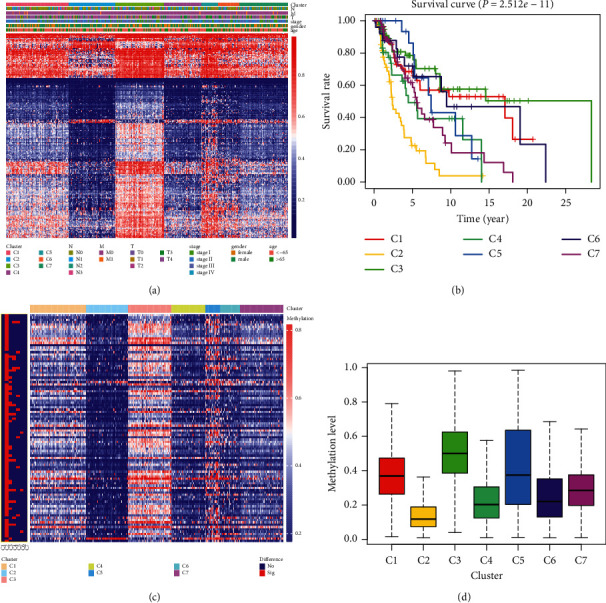
Comparison of clinical characteristics and methylation levels of different subtypes. (a) Clinical features of the seven melanoma subclusters and their methylation levels. (b) Survival curves of seven melanoma subtypes. (c) Overall heatmap of methylation sites that changed significantly in at least one subcluster compared with other subclusters. The significantly changed sites are indicated in red; other sites are marked in blue. (d) Comparison of the methylation levels of subcluster C2 with other subclusters. T, primary tumor; N, lymph node involvement; M, distant metastases; C, cluster; No, no statistical significance; Sig, statistical significance.

The study flow chart is shown in Figure 1. Considering the CDF (consensus cumulative distribution function)-consensus index graph and the delta area curve, we observed that when *k* = 7, there was a low variation coefficient and high consistency in the cluster graph, and the changes of the area under CDF curve were relatively small (Figures 2(a)–2(b)). Therefore, we divided the melanoma patients into seven subtypes. The seven subtypes were almost on a diagonal (Figure 2(c)), indicating there was good consistency. From the heatmap of the methylation levels for the seven subtypes, we found that the C2 subcluster had lower methylation levels than the other subclusters (Figure 3(a)). As shown in the survival curve in Figure 3(b), there was a statistical difference between the seven subclusters in terms of survival (*P* = 2.51 × 10^−11^) and the C2 subcluster had a lower survival rate than the others. These results indicated that these could distinguish patients with different prognostic status.

We further compared the clinical parameters of the different subclusters and found no significant differences in patient age and gender among the subclusters (Figure S1).

### 3.3. Comparison of the Methylation Levels of the Seven Methylation Subtypes

Comparison of the methylation levels of the 256 independent prognostic DNA methylation sites in the seven subclusters revealed that the methylation levels of DNA methylation sites in the C2 subcluster were lower than those of the other subclusters (Table S4, Figures 3(c)–3(d)). We identified 99 sites that had changed in at least one subtype compared with others, and most of these sites were present in the C2 subcluster (95 sites; Table S4, Figure 3(c)).

### 3.4. Construction of the Cox Prognostic Risk Regression Model and Detection Efficiency

As the C2 subtype had the lowest survival rate and methylation levels compared with other subclusters, and as this subcluster had the most significantly changed sites, we selected the significantly changed sites in the C2 subtype to draw the boxplot and construct the prognosis prediction model.

We used the coxph function in the R software to process the significantly changed methylation sites in the C2 subcluster and built the prediction model of clinical prognosis risk for patients with melanoma using the formula: risk score = Id1 × Co.1 + Id2 × Co.2 + Id3 × Co.3……+Idn × Co.n; the Id value and Co. are shown in Table 2. Using this prediction model, we calculated the risk scores for every patient and ranked them by the risk score (Table S5). The median risk score was −2.305 (Table S5).

**Figure 4:**
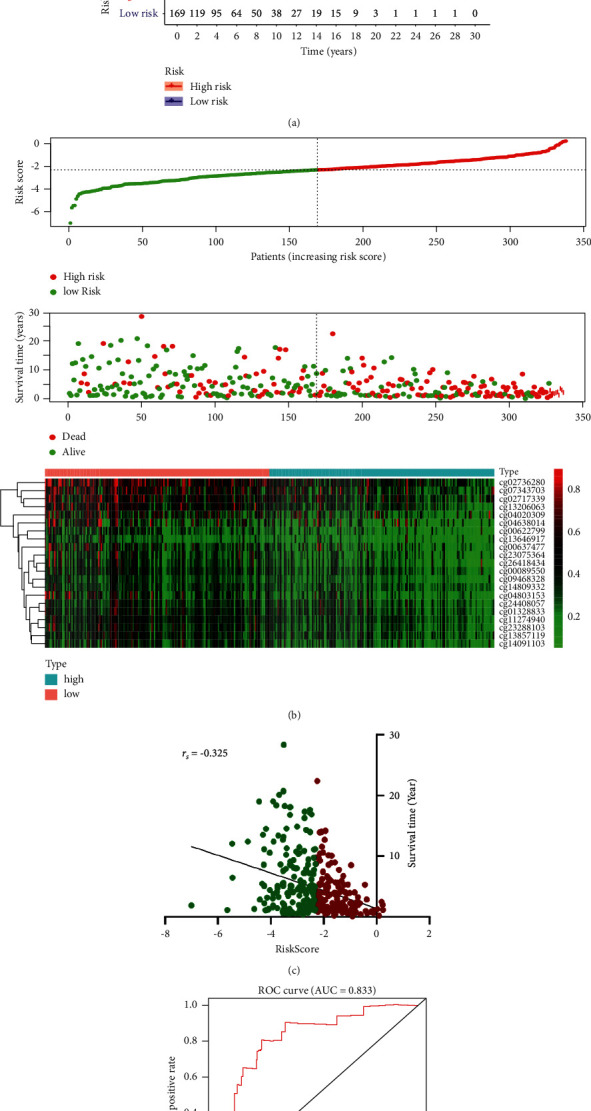
Cox risk regression model for melanoma prognosis assessment and detection efficiency. (a) Survival curve. (b) The patient survival status and methylation levels changed with the risk scores. (c) The correlation of patient risk scores and survival time. (d) The ROC curve of the constructed prognostic evaluation model. ROC, receiver operating characteristic; AUC, area under the curve; *r*_*s*_, coefficient of Spearman correlation analysis.

We then divided patients according to the median risk score: patients with a risk score higher than the median were high-risk cases, and those with lower risk scores than the median were low-risk cases. The survival rate of high-risk patients was significantly lower than that of patients with a low-risk score (*P* = 1.11 × 10^−15^, Figure 4(a)). With the increasing of the risk score, the number of patients with melanoma increased and the methylation levels of the sites in the risk prediction model decreased (Figure 4(b)). There was a negative correlation between patient survival time and risk scores, and patients in the high-risk group had poor prognosis (*r*_*s*_ = −0.325, 95% confidence interval, −0.420 to 0.224, *P* < 0.0001, Figure 4(c)). These results indicate that the risk score obtained from this risk prediction model could predict the prognosis of patients with melanoma.

We further used ROC (receiver operating characteristic) curve analysis to evaluate the efficiency of this method and found that the method had high efficiency. Area under the curve (AUC) was 0.833 (*P* < 0.05), demonstrating that this model could distinguish patients in the high-risk group from patients in the low-risk group (Figure 4(d)). Then, we randomly selected 60% of the samples to test the prediction model, we did this for 100 times, the mean AUC was 0.82, and the *P* value for the comparison of survival probabilities (high-risk group *vs.* low-risk group) was less than 0.05 in almost all the 100 trials (96%, Table S6), which confirmed the test efficiency of the prognosis prediction model we built in this research.

### 3.5. Pathway Enrichment of Genes Corresponding to the Prognostic Methylation Sites

**Figure 5:**
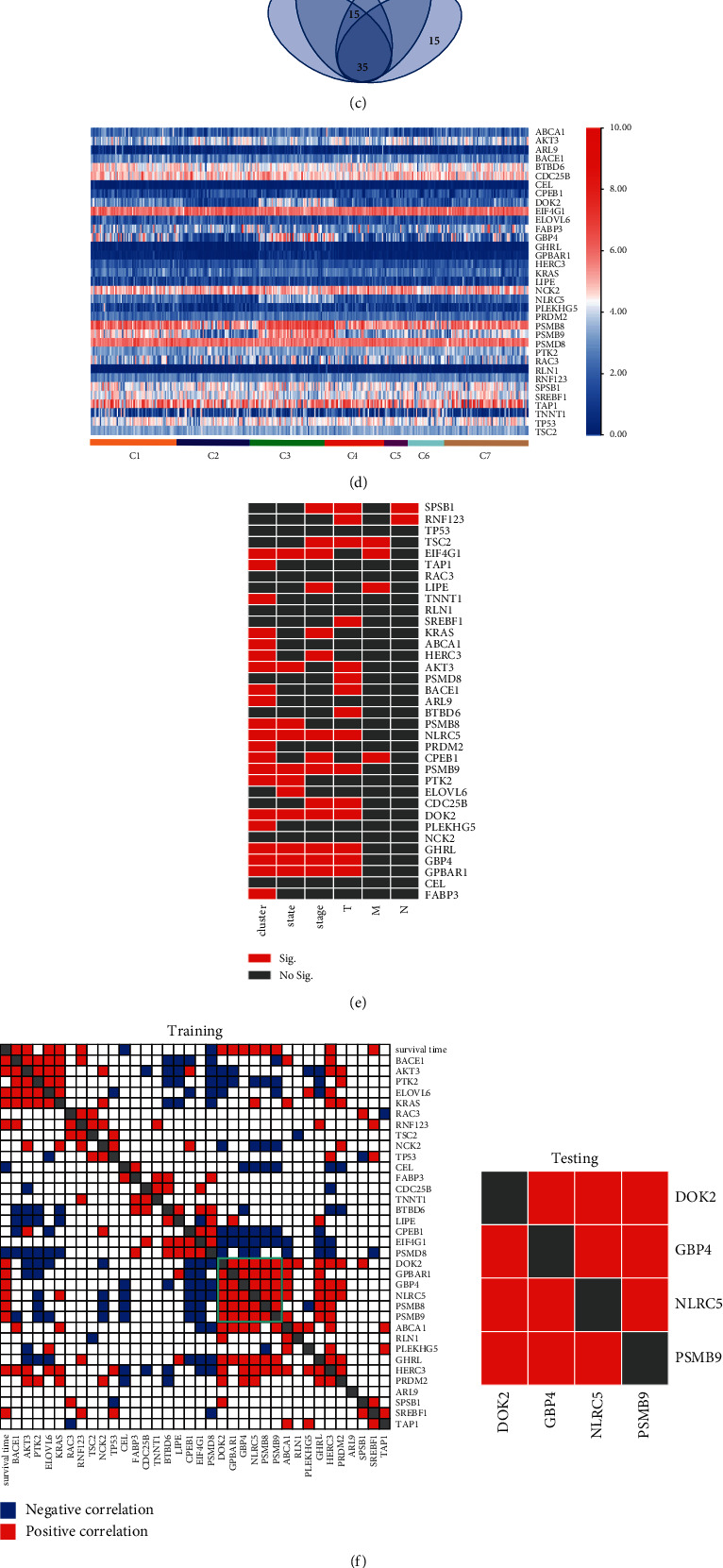
Pathway enrichment of genes associated with independent prognostic methylation sites and the detailed analysis of key genes. (a) Network of enriched pathways. The width of the line represents the Kappa score between two connected nodes. If the Kappa score increased, the width of the line is wider. (b) The number of genes in different KEGG pathways and −log (*P* value). (c) The Venn diagram of the top 50 genes in the four scoring methods. Among the 50 genes, 35 genes were included in all four scoring methods. (d) Heatmap of the expression levels of 35 hub genes in each subgroup. (e) The comparison of the levels of 35 key genes in different clinical groups (*P* value). Red indicates *P* <0.05; and gray indicates not significant. (f) Correlation of the expression of hub genes or the patient survival time in the training group and verification in the testing group. (g) The heatmap of the expression levels of the critical corresponding genes selected from (f) and their DNA methylation sites in different clinical groups. MCC, maximal clique centrality; MNC, maximum neighborhood component; EPC, edge percolated component; C, cluster; T, primary tumor; N, lymph node involvement; M, distant metastases; Sig, significant difference, *P* < 0.05; No Sig., no significant difference, *P* > 0.05.

Our pathway enrichment results of genes corresponding to the prognostic-related methylation sites are shown in Table S7 and Figures 5(a)–5(b). The results identified pathways such as choline metabolism in cancer, colorectal cancer, and melanoma. The correlation between the pathways is shown in Figure 5(a); the pathways were focused on two groups, i.e., autoimmunity and immune-related pathways and cancer and its related pathways.

We next scored genes in the significant pathways using the CytoHubba plug-in of Cytoscape software. We used four methods to score the genes and selected the top 50 genes in every method to draw a Venn diagram. We identified 35 genes among the top 50 genes in all of the four methods (Figure 5(c)) and selected these 35 genes as the hub genes.

The expression levels of the hub genes in the seven subgroups are shown in Figure 5(d). We integrated the expression of the 35 hub genes with patient information and found that the gene expressions of docking protein 2 (DOK2), *G* protein-coupled bile acid receptor 1 (GPBAR1), guanylate-binding protein 4 (GBP4), proteasome 20S subunit beta 9 (PSMB9), and NLR family CARD domain containing 5 (NLRC5) were significantly altered in different DNA methylation subgroups, patients with different survival status, different stages, and T categories (Figure 5(e), Table 3, *P* < 0.05). To explore the correlation of the 35 hub genes, we conducted Pearson correlation analysis and found that there was positive correlation between DOK2, GPBAR1, GBP4, PSMB9, and NLRC5; further, we validated their correlation in the testing group (GSE98394) and confirmed the positive correlation between DOK2, GBP4, PSMB9, and NLRC5 (*P* < 0.05, Tables S8-S9, Figure 5(f)). Then, we searched the independent prognosis-associated methylation sites on their promoters' regions and found cg00533183 and cg07156249 were on the promoters' of PSMB9, cg07839457 was on NLRC5, cg21163717 was on DOK2, and cg27285720 was on GBP4, the methylation levels of those sites were higher in dead than the survival patients, patients in stages II ∼ IV than stage I, patients in T2∼4 than T0∼1, and the gene expression of their corresponding genes had an opposite trend to them just as we expected (Figure 5(g)).

## 4. Discussion

Melanoma is a malignant cancer with an increasing incidence worldwide [2]. A patient diagnosed at stage IV (based on American Cancer Federation Staging) has very few treatment options and a predicted survival of less than 2 years [2, 27, 28]. Therefore, identifying methods for early diagnosis and developing more effective treatment strategies for patients are critical.

The pathogenesis of melanoma is mediated by a series of genetic and epigenetic changes [2]. Epigenetic modifications silence the expression of melanin-related genes [27, 29]. Aberrant DNA methylation is an epigenetic hallmark of melanoma and plays critical roles in the formation and progression of melanoma [30]. Changes in DNA methylation sites in tumor suppressor genes are found in patients with metastatic melanoma [31]. Therefore, in this study, we performed an in-depth analysis of DNA methylation in melanoma to better understand the molecular mechanism of melanoma and potentially identify key sites that may be new diagnostic markers or therapeutic treatment targets.

In this study, we successfully built a prognostic risk assessment model based on the independent prognosis-associated DNA methylation sites, and this model could efficiently distinguish low-risk patient from high-risk patients (Figure 4, Table S6). Patients with a high-risk score showed a low survival probability, and there was significant negative correlation between the risk score and patient survival time (Figures 4(a)–4(c)). This indicated that this model could be used to predict the prognosis of patients with melanoma and thus provides a new method for doctors to evaluate patient prognosis and provide appropriate personalized treatments. Research has shown that abnormal DNA methylation changes occur before the disordered translation of the protein [32–34]. Therefore, the predicted model built with these prognosis-related DNA methylation sites may be useful for early diagnosis.

The enriched pathways of genes corresponding to the prognostic methylation sites were mainly in autoimmune or immune-related disease and tumor-related pathways. There was a correlation between the melanoma pathway and pathways such as glioma and colorectal cancer. The tumor pathways may be connected to each other through signaling pathways such as the central carbon metabolism, phospholipase D signaling, or sphingolipid signaling pathways. Alternatively, they may affect the immune homeostasis of melanoma patients and further contribute to the development of this disease by pathways such as the B cell receptor signaling pathway (Figures 5(a)– and 5(b)). The immune-related pathways were mainly concentrated on the natural killer cell-mediated cytotoxicity, antigen processing, and presentation pathways, suggesting that the independent prognosis-related DNA methylation sites may affect the expression of genes encoding key factors that influence the immune function, resulting in abnormal activities of natural killer or antigen-presenting cells in melanoma patients. These alterations may affect the development of the disease and impact the prognosis of the patient.

Further, we found that the gene expression levels of DOK2, GBP4, PSMB9, and NLRC5 were changed according to different methylation subclusters, patient state, tumor stage, and clinical T categories; the transcript level of PSMB8 was changed in different methylation subgroups and patients with different survival states (Figures 5(d)––5(e)). DOK2 is an aptamer protein that regulates the tyrosine kinase signaling pathway, including tyrosine kinase receptors such as epidermal growth factor receptors [35]. The expression level of DOK2 was decreased in gastric cancer, indicating that DOK2 may be a potential tumor suppressor in solid tumors [36]. Based on the abnormal expression of DOK2 in digestive tract tumors such as gastric and colorectal cancers, some researchers established a prognostic evaluation model including DOK2 that effectively identified patients with poor prognosis [36, 37]. In addition, DOK2 deficiency in ovarian cancer induced carboplatin resistance by inhibiting the apoptosis of tumor cells [38], while the expression of DOK2/Ras p21 protein activator 1 was associated with prognosis and quality of life of breast cancer patients. The deficiency of these proteins may result in tumor enlargement or progression and lymph node metastasis [39]. Deletion of DOK2 in a mouse model led to the accelerated formation of lung tumors [40]. GBP4 is a guanylate-binding protein that is involved in pathological processes such as tumor formation and progression. A prognosis predictive model that was constructed using GBP4 was used to evaluate the prognosis of melanoma patients [41, 42]. A high expression of GPB4 was correlated with the favorable overall survival in skin cutaneous melanoma patients for more than 30 years [42]. Proteasome 20S subunit beta 8/9 (PSMB8/9) proteins are critical immune proteasome subunits. PSMB8/9 overexpression indicated a good prognosis in patients with melanoma and a good response to immune-checkpoint inhibitors [43]. Increased expression of NLRC5 was associated with the slower growth of the tumor in a mouse melanoma model and prolonged survival time in patients with melanoma [44]. NLRC5 was considered a potential immune molecule in antitumor treatment that could be used to improve tumor immunogenicity and restore antitumor immunity [45]. As the above description, DOK2, GBP4, PSMB9, and NLRC5 were tumor suppressors, in this research, their expressions were decreased in patients with poor prognosis and the levels of the methylation sites on their promoter regions were increased (Figure 5(g)), the latter might hinder the expression of the former. The weak expressions of those tumor suppressors could promote the complicated formation or the development of melanoma.

Furthermore, we found a positive correlation between the gene expression levels of DOK2, GBP4, PSMB9, and NLRC5, and their correlation had been confirmed in the testing group (Figure 5(f)), indicating that they may have a cooperative relationship. The expression of one gene might drive the expression of other genes to form a positively regulated cluster. In addition, the previously reported critical functions of these genes and encoded proteins in various cancers including melanoma help support and validate our findings. We speculate that the alterations in the independent prognosis-related methylation sites affect the expression of the corresponding genes, especially the genes in this cluster (Figures 5(f) and 5(g)), and therefore influence the development or progression of melanoma. The positive relationships between these genes indicate they may be coordinately involved in melanoma development.

There are also some limitations in this research, which are as follows: we used 475 methylation files to build the predicted model of patients' prognosis and found the independent prognosis sites' corresponding genes as *DOK2*, *GBP4*, *PSMB9*, and *NLRC5* were critical and positively correlated with each other, which was validated in the testing group, but there are few data sets to be found to retest the efficiency of the predicted model. In the future, we will reexamine them clinically.

## 5. Conclusion

Here, we established a prediction model for the prognosis of patients with melanoma based on prognosis-related DNA methylation sites in gene promoter regions. This model efficiently distinguished high-risk patient from low-risk patients. Among the 35 hub genes corresponding to the prognosis-related DNA methylation sites, the gene expression levels of DOK2, GBP4, PSMB9, and NLRC5 were significantly changed in different patient subgroups according to DNA methylation subtypes, patient states, tumor stages, and T categories. The genes were positively correlated with each other, and the altered DNA methylation levels on the gene promoter regions of these genes might affect their expression. Furthermore, these genes might be involved in the pathological process of melanoma. We plan to focus future studies on these five identified hub genes and explore their potential mechanisms in DNA methylation and their interactions. Our findings may help provide new targets for clinicians to treat patients with melanoma and enable early diagnosis based on these critical DNA methylation sites.

## Figures and Tables

**Table 1:** Patient information.

Features		Classification	No.	Comparison	
				Statis.	*P* value
Gender		Female	180	7.935^∗∗^	0.243
		Male	290		
Age (years)		≤60	250	5.526^∗^	0.478
		>60	212		
State		Dead	211	25.031^∗∗^	<0.0001
		Alive	259		
Survival (years)		<1	63	27.929^∗^	<0.0001
		1–5	246		
		>5	151		
TNM	T	T0	23	36.324^∗∗^	0.051
		T1	42		
		T2	78		
		T3	90		
		T4	153		
	M	M0	418	6.640^∗∗^	0.355
		M1	24		
	N	N0	235	17.659^∗∗^	0.478
		N1	74		
		N2	49		
		N3	55		
Stage		0	7	25.909^∗∗^	0.102
		I	77		
		II	140		
		III	171		
		IV	23		

^∗^Kruskal–Wallis test; ^∗∗^Chi-test; No., patient number; Statis., statistics; T, primary tumor; N, lymph node involvement; M, distant metastases.

**Table 2:** Coefficients in the model for prognosis prediction.

Id	Co.	Id	Co.	Id	Co.
cg00089550	5.075	cg09468328	−3.736	cg04638014	−0.948
cg00622799	−1.955	cg11274940	−7.434	cg04803153	−2.456
cg00637477	−1.156	cg13206063	−2.145	cg07343703	−1.330
cg01328833	−4.145	cg13646917	−2.341	cg23075364	3.615
cg02717339	2.294	cg13857119	1.973	cg23288103	4.650
cg02736280	−2.709	cg14091103	3.246	cg24408057	5.825
cg04020309	−0.930	cg14809332	4.071	cg26418434	−6.482

Id, methylation site identification; Co., coefficient.

**Table 3:** Comparison of the expression levels of 35 hub genes among different clinical groups (*P* values are shown).

Genes	Cluster	State	Stage	T	M	N
SPSB1	0.63	0.285	0.024	0.044	0.908	0.009
RNF123	0.507	0.54	0.27	0.041	0.224	0.012
TP53	0.764	0.315	0.054	0.734	0.718	0.062
TSC2	0.191	0.715	0.019	0.009	0.043	0.068
EIF4G1	0.012	0.039	0.026	0.923	0.007	0.103
TAP1	0.034	0.06	0.875	0.352	0.854	0.13
RAC3	0.834	0.288	0.283	0.713	0.075	0.15
LIPE	0.325	0.406	0.035	0.24	0.016	0.199
TNNT1	0.022	0.938	0.094	0.197	0.16	0.217
RLN1	0.635	0.332	0.743	0.836	0.826	0.24
SREBF1	0.18	0.257	0.209	0.026	0.701	0.253
KRAS	0.024	0.529	0.035	0.186	0.098	0.276
ABCA1	0	0.053	0.49	0.359	0.773	0.298
HERC3	0.004	0.515	0.047	0.348	0.322	0.306
AKT3	0.003	0.021	0.417	0.026	0.356	0.312
PSMD8	0.096	0.237	0.076	0.006	0.431	0.313
BACE1	0.006	0.125	0.468	0.039	0.368	0.341
ARL9	0	0.154	0.589	0.542	0.193	0.38
BTBD6	0.195	0.886	0.065	0.008	0.758	0.393
PSMB8	0	0	0.066	0.242	0.758	0.435
NLRC5	0	0	0.001	0.004	0.435	0.448
PRDM2	0.026	0.188	0.097	0.161	0.083	0.456
CPEB1	0.027	0.474	0.001	0.216	0.044	0.485
PSMB9	0	0	0.003	0.021	0.878	0.487
PTK2	0	0.049	0.462	0.147	0.196	0.497
ELOVL6	0.102	0.043	0.448	0.076	0.804	0.512
CDC25 B	0.54	0.062	0.01	0.001	0.943	0.533
DOK2	0	0.008	0.001	0.003	0.802	0.587
PLEKHG5	0	0.842	0.829	0.66	0.556	0.6
NCK2	0.23	0.344	0.659	0.906	0.329	0.625
GHRL	0	0.013	0.031	0.001	0.095	0.727
GBP4	0	0.002	0	0	0.203	0.736
GPBAR1	0	0.005	0.006	0.041	0.994	0.841
CEL	0.05	0.234	0.449	0.561	0.763	0.849
FABP3	0.014	0.993	0.234	0.291	0.844	0.856

## Data Availability

The data that support the findings of this study are available from the corresponding author or the first author upon reasonable request.

## Abbreviations

TNM: Primary tumor, lymph node involvement, distant metastases
DNMT: DNA methyltransferase
CpG: 5′-Cytosine-phosphate-guanine-3'
TCGA: The Cancer Genome Atlas
UCSC: University of California Santa Cruz
MCC: Maximal clique centrality
EPC: Edge percolated component
MNC: Maximum neighborhood component
CDF: Consensus cumulative distribution function
r_s_: Coefficient of Spearman correlation analysis
ROC: Receiver operating characteristic
AUC: Area under the curve
DOK2: Docking protein 2
GPBAR1: *G* protein-coupled bile acid receptor 1
GBP4: Guanylate-binding protein 4
PSMB: Proteasome 20S subunit beta
NLRC5: NLR family CARD domain containing 5
No.: Patient number
Statis.: Statistics
Id: Methylation site's identification
Co.: Coefficient
C: Cluster
G: Grade
No Sig.: No significant difference
Sig: There was significance statistically.

## Supplementary Materials

Supplementary Materials
*Supplementary Table 1.* Prognosis-related methylation sites by univariate Cox regression analysis (783 sites were found, *P* < 0.0001). *Supplementary Table 2*. Multivariate Cox regression analysis of the 783 methylation sites (256 sites were found, *P* < 0.0001). *Supplementary Table 3.* Level of the 256 sites in 338 samples and the follow-up. *Supplementary Table 4*. Analysis of differences in methylation site levels between the 7 clusters. *Supplementary Table 5.* Risk assessment. *Supplementary Table 6*. Testing the prediction model in 60% of the samples (randomly) for 100 times. *Supplementary Table 7*. Functional enrichment analysis of genes and 28 pathways were observed. *Supplementary Table 8*. The correlation coefficients between the expression levels of the 35 hub genes (^*∗∗*^*P* < 0.01, ^*∗*^*P* < 0.05). *Supplementary Table 9.* The correlation coefficients of the critical genes validated in the testing group (^*∗∗*^*P* < 0.01). *Figure S1*. The clinical features in different DNA methylation subgroups. The clinical T categories (a), N stage (b), and M status (c) of different subtypes. The tumor stage (d), patient ages (e), and gender (f) in different subtypes. C, cluster; T, primary tumor; N, lymph node involvement; M, distant metastases.
