# Supplementary material for: Identification of Melanoma Subsets Based on DNA Methylation Sites and Construction of a Prognosis Evaluation Model
Source: J Oncol. 2022 Oct 11;2022:6608650. doi: 10.1155/2022/6608650 (PMC9578801; doi:10.1155/2022/6608650)
Supplement: Supplementary Materials — Supplementary Table 1. Prognosis-related methylation sites by univariate Cox regression analysis (783 sites were found, P < 0.0001). Supplementary Table 2. Multivariate Cox regression analysis of the 783 methylation sites (256 sites were found, P < 0.0001). Supplementary Table 3. Level of the 256 sites in 338 samples and the follow-up. Supplementary Table 4. Analysis of differences in methylation site levels between the 7 clusters. Supplementary Table 5. Risk assessment. Supplementary Table 6. Testing the prediction model in 60% of the samples (randomly) for 100 times. Supplementary Table 7. Functional enrichment analysis of genes and 28 pathways were observed. Supplementary Table 8. The correlation coefficients between the expression levels of the 35 hub genes (∗∗P < 0.01, ∗P < 0.05). Supplementary Table 9. The correlation coefficients of the critical genes validated in the testing group (∗∗P < 0.01). Figure S1. The clinical features in different DNA methylation subgroups. The clinical T categories (a), N stage (b), and M status (c) of different subtypes. The tumor stage (d), patient ages (e), and gender (f) in different subtypes. C, cluster; T, primary tumor; N, lymph node involvement; M, distant metastases. [file 6608650.f1.zip › Supplementary Table 1. Prognosis-related methylation sites by Univariate Cox regression analysis .docx]

| **Supplementary Table 1. Prognosis-related methylation sites by Univariate Cox regression analysis (783 sites were found, *P*<0.0001).** | | | | |
| --- | --- | --- | --- | --- |
| **ID** | **HR** | **Low.95% CI** | **High.95%CI** | **pvalue** |
| cg09812541 | 0.011282 | 0.001178 | 0.108015 | 9.99E-05 |
| cg00152411 | 0.018903 | 0.002566 | 0.139269 | 9.83E-05 |
| cg11694510 | 9.531036 | 3.065016 | 29.6379 | 9.82E-05 |
| cg24555065 | 19514513 | 4186.933 | 9.1E+10 | 9.82E-05 |
| cg26108594 | 0.001121 | 3.68E-05 | 0.034198 | 9.78E-05 |
| cg12866938 | 8.77E+20 | 2.55E+10 | 3.01E+31 | 9.78E-05 |
| cg16967626 | 8.864433 | 2.958053 | 26.56415 | 9.75E-05 |
| cg25251478 | 0.071361 | 0.018914 | 0.269231 | 9.75E-05 |
| cg10145584 | 31.19938 | 5.530093 | 176.019 | 9.73E-05 |
| cg23075364 | 0.156414 | 0.061539 | 0.397561 | 9.7E-05 |
| cg16696476 | 0.086245 | 0.025163 | 0.2956 | 9.65E-05 |
| cg18442019 | 0.24136 | 0.118161 | 0.493009 | 9.59E-05 |
| cg07629007 | 0.238653 | 0.116179 | 0.490237 | 9.59E-05 |
| cg11824827 | 69.41802 | 8.247102 | 584.3096 | 9.57E-05 |
| cg16553052 | 0.222263 | 0.104423 | 0.473085 | 9.54E-05 |
| cg18569734 | 6.123192 | 2.466427 | 15.20153 | 9.39E-05 |
| cg24670552 | 0.146016 | 0.05561 | 0.383394 | 9.37E-05 |
| cg02091185 | 0.269025 | 0.139222 | 0.519852 | 9.37E-05 |
| cg14452720 | 31916445 | 5484.037 | 1.86E+11 | 9.36E-05 |
| cg02593884 | 0.144199 | 0.054576 | 0.380997 | 9.36E-05 |
| cg20569108 | 0.092485 | 0.028022 | 0.305238 | 9.31E-05 |
| cg05029558 | 0.040476 | 0.008105 | 0.202141 | 9.29E-05 |
| cg24287218 | 5.072 | 2.246752 | 11.44994 | 9.29E-05 |
| cg06311355 | 0.08695 | 0.025555 | 0.295839 | 9.25E-05 |
| cg26617637 | 0.161268 | 0.064616 | 0.402492 | 9.22E-05 |
| cg20930114 | 0.000985 | 3.07E-05 | 0.031656 | 9.22E-05 |
| cg25591888 | 0.171328 | 0.070764 | 0.41481 | 9.21E-05 |
| cg24124145 | 0.018942 | 0.002595 | 0.138289 | 9.21E-05 |
| cg05059480 | 0.100317 | 0.031696 | 0.317504 | 9.17E-05 |
| cg21006031 | 0.193702 | 0.085117 | 0.440812 | 9.14E-05 |
| cg06805940 | 0.120305 | 0.041644 | 0.347546 | 9.13E-05 |
| cg08888354 | 0.100904 | 0.031984 | 0.318332 | 9.13E-05 |
| cg23285459 | 0.337908 | 0.196227 | 0.581886 | 9.13E-05 |
| cg24408057 | 0.121008 | 0.042024 | 0.348442 | 9.09E-05 |
| cg12221864 | 0.241371 | 0.118455 | 0.49183 | 9.08E-05 |
| cg01335785 | 0.001725 | 7.13E-05 | 0.041728 | 9.08E-05 |
| cg01804934 | 3.801484 | 1.947801 | 7.41928 | 9.07E-05 |
| cg11683242 | 31.91043 | 5.634927 | 180.7079 | 9.07E-05 |
| cg12433575 | 0.221458 | 0.104109 | 0.471081 | 9.06E-05 |
| cg23762517 | 0.104907 | 0.033937 | 0.324292 | 9.02E-05 |
| cg13876650 | 0.033944 | 0.006243 | 0.184572 | 9.01E-05 |
| cg08767286 | 0.110248 | 0.036573 | 0.332334 | 8.98E-05 |
| cg06200697 | 0.099197 | 0.031214 | 0.315248 | 8.97E-05 |
| cg00683665 | 17730.87 | 132.7135 | 2368891 | 8.96E-05 |
| cg11769290 | 0.000102 | 1.03E-06 | 0.010137 | 8.92E-05 |
| cg10613224 | 0.308651 | 0.171445 | 0.555661 | 8.9E-05 |
| cg01681009 | 1.7E+26 | 1.29E+13 | 2.23E+39 | 8.89E-05 |
| cg15837913 | 3.953512 | 1.988164 | 7.861651 | 8.88E-05 |
| cg20552752 | 6296.34 | 79.31049 | 499856.9 | 8.87E-05 |
| cg26742440 | 1.02E+11 | 320274.6 | 3.24E+16 | 8.82E-05 |
| cg13500819 | 14.64468 | 3.828768 | 56.0145 | 8.8E-05 |
| cg00168785 | 0.00972 | 0.000959 | 0.098457 | 8.78E-05 |
| cg14407437 | 0.053722 | 0.012465 | 0.231523 | 8.75E-05 |
| cg03760308 | 0.188989 | 0.082228 | 0.434363 | 8.71E-05 |
| cg19702802 | 25.22247 | 5.032328 | 126.4173 | 8.68E-05 |
| cg26256192 | 0.254253 | 0.128334 | 0.50372 | 8.64E-05 |
| cg14550760 | 0.105865 | 0.034509 | 0.324764 | 8.62E-05 |
| cg24049888 | 6.598818 | 2.573081 | 16.92305 | 8.61E-05 |
| cg12024906 | 0.072653 | 0.019637 | 0.268809 | 8.56E-05 |
| cg02402423 | 0.134063 | 0.049197 | 0.365322 | 8.54E-05 |
| cg15598244 | 0.00672 | 0.000554 | 0.081509 | 8.54E-05 |
| cg22436229 | 0.000398 | 8.02E-06 | 0.019777 | 8.53E-05 |
| cg14754555 | 0.175682 | 0.073796 | 0.418236 | 8.5E-05 |
| cg07817320 | 177910.4 | 428.3669 | 73890173 | 8.5E-05 |
| cg03884592 | 0.034459 | 0.006425 | 0.184819 | 8.49E-05 |
| cg20248866 | 0.088696 | 0.0265 | 0.296867 | 8.48E-05 |
| cg08383160 | 0.176243 | 0.074161 | 0.418843 | 8.48E-05 |
| cg02954987 | 0.114838 | 0.039033 | 0.337864 | 8.47E-05 |
| cg00066663 | 0.009481 | 0.00093 | 0.096632 | 8.4E-05 |
| cg25856663 | 0.045653 | 0.009806 | 0.21254 | 8.38E-05 |
| cg23244095 | 0.127998 | 0.045955 | 0.356508 | 8.37E-05 |
| cg23857896 | 0.029955 | 0.005218 | 0.17195 | 8.33E-05 |
| cg12749636 | 1.48E-05 | 5.83E-08 | 0.003751 | 8.24E-05 |
| cg10601943 | 6.422409 | 2.54478 | 16.20861 | 8.23E-05 |
| cg23181159 | 0.221749 | 0.10478 | 0.469298 | 8.22E-05 |
| cg02851047 | 0.10768 | 0.035515 | 0.32648 | 8.22E-05 |
| cg18131548 | 6.201112 | 2.500777 | 15.37674 | 8.21E-05 |
| cg17001035 | 0.044959 | 0.009602 | 0.210506 | 8.21E-05 |
| cg18183774 | 0.233184 | 0.112988 | 0.481242 | 8.2E-05 |
| cg26449680 | 0.045738 | 0.009855 | 0.212275 | 8.18E-05 |
| cg09874127 | 8.959057 | 3.009232 | 26.67282 | 8.18E-05 |
| cg12262564 | 0.012462 | 0.001407 | 0.110394 | 8.15E-05 |
| cg03780927 | 0.089842 | 0.027096 | 0.297886 | 8.14E-05 |
| cg24062767 | 0.000527 | 1.24E-05 | 0.022519 | 8.13E-05 |
| cg19374752 | 0.06499 | 0.016693 | 0.253027 | 8.1E-05 |
| cg04013166 | 0.144286 | 0.055104 | 0.377801 | 8.08E-05 |
| cg17078393 | 32.76461 | 5.784122 | 185.5976 | 8.03E-05 |
| cg03299838 | 0.000492 | 1.12E-05 | 0.021653 | 8E-05 |
| cg18221580 | 0.109738 | 0.036604 | 0.328998 | 8E-05 |
| cg04992930 | 1.27E-11 | 4.89E-17 | 3.29E-06 | 7.98E-05 |
| cg11611856 | 0.27386 | 0.143908 | 0.521163 | 7.98E-05 |
| cg20317748 | 0.112278 | 0.037885 | 0.332751 | 7.98E-05 |
| cg13282195 | 0.051749 | 0.011884 | 0.225343 | 7.98E-05 |
| cg14809332 | 0.061424 | 0.01536 | 0.24563 | 7.97E-05 |
| cg06795963 | 0.128725 | 0.04649 | 0.356423 | 7.97E-05 |
| cg13548265 | 0.009972 | 0.001011 | 0.098387 | 7.97E-05 |
| cg07695566 | 0.176765 | 0.074739 | 0.418068 | 7.96E-05 |
| cg27629145 | 0.120614 | 0.042185 | 0.344857 | 7.94E-05 |
| cg24435209 | 0.103225 | 0.033417 | 0.318861 | 7.94E-05 |
| cg14599956 | 0.000962 | 3.05E-05 | 0.030298 | 7.94E-05 |
| cg17427615 | 0.121383 | 0.04259 | 0.345948 | 7.93E-05 |
| cg21993406 | 0.086274 | 0.02555 | 0.291322 | 7.93E-05 |
| cg20242781 | 0.223965 | 0.106534 | 0.470836 | 7.92E-05 |
| cg11934419 | 0.137445 | 0.051303 | 0.368226 | 7.92E-05 |
| cg15287850 | 0.199608 | 0.089689 | 0.444239 | 7.89E-05 |
| cg26444282 | 0.21907 | 0.103094 | 0.465512 | 7.87E-05 |
| cg01789576 | 0.10559 | 0.034593 | 0.322296 | 7.86E-05 |
| cg16859636 | 0.238544 | 0.117133 | 0.485799 | 7.83E-05 |
| cg12461287 | 0.046851 | 0.01026 | 0.213943 | 7.81E-05 |
| cg23967461 | 0.008015 | 0.000731 | 0.087896 | 7.81E-05 |
| cg16121744 | 0.127057 | 0.04565 | 0.353637 | 7.81E-05 |
| cg15582789 | 0.021135 | 0.003119 | 0.143222 | 7.8E-05 |
| cg00605777 | 0.132659 | 0.048708 | 0.361304 | 7.77E-05 |
| cg23851932 | 60.84123 | 7.932126 | 466.6662 | 7.74E-05 |
| cg27506609 | 0.221724 | 0.10505 | 0.467982 | 7.74E-05 |
| cg04650656 | 0.240774 | 0.118838 | 0.487828 | 7.74E-05 |
| cg01617074 | 0.052605 | 0.012213 | 0.226586 | 7.73E-05 |
| cg01791634 | 6.942082 | 2.65775 | 18.13282 | 7.64E-05 |
| cg11383747 | 1.7E+08 | 14239.18 | 2.03E+12 | 7.59E-05 |
| cg18607308 | 0.025914 | 0.004243 | 0.158254 | 7.59E-05 |
| cg16440442 | 0.226573 | 0.108618 | 0.472623 | 7.56E-05 |
| cg06697251 | 0.118696 | 0.041317 | 0.340993 | 7.55E-05 |
| cg12537329 | 0.214443 | 0.100047 | 0.459644 | 7.55E-05 |
| cg26546862 | 0.012379 | 0.001407 | 0.10892 | 7.55E-05 |
| cg05851542 | 0.120532 | 0.042278 | 0.343633 | 7.55E-05 |
| cg06529685 | 18.47862 | 4.36315 | 78.25988 | 7.48E-05 |
| cg25766774 | 0.118361 | 0.04117 | 0.340279 | 7.47E-05 |
| cg23696248 | 0.162543 | 0.066158 | 0.399349 | 7.45E-05 |
| cg02114346 | 0.123399 | 0.043827 | 0.347444 | 7.45E-05 |
| cg22151713 | 0.235226 | 0.114955 | 0.48133 | 7.45E-05 |
| cg14557185 | 0.103936 | 0.033909 | 0.318581 | 7.45E-05 |
| cg13449463 | 0.168644 | 0.069911 | 0.406816 | 7.44E-05 |
| cg06808498 | 3.353751 | 1.843205 | 6.102219 | 7.42E-05 |
| cg05295388 | 0.225046 | 0.107631 | 0.470549 | 7.4E-05 |
| cg16990174 | 0.124517 | 0.044465 | 0.348687 | 7.33E-05 |
| cg22331349 | 0.105698 | 0.034829 | 0.320773 | 7.27E-05 |
| cg04905421 | 0.231366 | 0.112299 | 0.476675 | 7.22E-05 |
| cg00983520 | 0.066405 | 0.017404 | 0.253362 | 7.2E-05 |
| cg01732984 | 0.033322 | 0.006213 | 0.178704 | 7.2E-05 |
| cg19443920 | 0.188634 | 0.08279 | 0.429794 | 7.19E-05 |
| cg06380725 | 0.163665 | 0.066972 | 0.399963 | 7.19E-05 |
| cg24596836 | 22443557 | 5281.217 | 9.54E+10 | 7.16E-05 |
| cg02473395 | 6.12E+15 | 98994582 | 3.78E+23 | 7.14E-05 |
| cg02055253 | 0.148492 | 0.057937 | 0.380583 | 7.14E-05 |
| cg12984086 | 0.115029 | 0.039573 | 0.334357 | 7.12E-05 |
| cg11862081 | 0.144535 | 0.055657 | 0.375343 | 7.11E-05 |
| cg07103618 | 0.167594 | 0.069428 | 0.40456 | 7.11E-05 |
| cg13374901 | 0.100777 | 0.032484 | 0.312643 | 7.1E-05 |
| cg24107665 | 0.02635 | 0.004382 | 0.158439 | 7.1E-05 |
| cg10880485 | 4.98852 | 2.257948 | 11.02121 | 7.07E-05 |
| cg19583655 | 0.109427 | 0.036745 | 0.325877 | 7.07E-05 |
| cg02266500 | 0.087119 | 0.026147 | 0.290266 | 7.06E-05 |
| cg05044414 | 0.120653 | 0.042526 | 0.342312 | 7.04E-05 |
| cg04717699 | 0.204365 | 0.093413 | 0.447101 | 7.03E-05 |
| cg09951047 | 0.286651 | 0.154816 | 0.530753 | 7.03E-05 |
| cg25687894 | 0.003328 | 0.0002 | 0.05542 | 7.01E-05 |
| cg01601628 | 3.542379 | 1.899069 | 6.607683 | 7E-05 |
| cg02245810 | 0.211312 | 0.098228 | 0.454583 | 6.98E-05 |
| cg18621299 | 4.44966 | 2.132233 | 9.285795 | 6.97E-05 |
| cg24915511 | 0.008957 | 0.000878 | 0.091424 | 6.94E-05 |
| cg02953960 | 0.041927 | 0.008788 | 0.200039 | 6.94E-05 |
| cg18213545 | 0.06312 | 0.016185 | 0.246159 | 6.93E-05 |
| cg05593775 | 0.14947 | 0.058606 | 0.381211 | 6.92E-05 |
| cg04552852 | 0.081291 | 0.023613 | 0.279853 | 6.92E-05 |
| cg20000342 | 0.006603 | 0.000557 | 0.078256 | 6.9E-05 |
| cg26288449 | 0.102799 | 0.03353 | 0.315171 | 6.89E-05 |
| cg07960067 | 9.54E-27 | 1.47E-39 | 6.2E-14 | 6.88E-05 |
| cg07168392 | 1.58E+16 | 1.67E+08 | 1.49E+24 | 6.86E-05 |
| cg08414643 | 6.206922 | 2.527014 | 15.24562 | 6.84E-05 |
| cg10950111 | 0.23833 | 0.117685 | 0.482653 | 6.8E-05 |
| cg07617759 | 0.19181 | 0.085123 | 0.43221 | 6.78E-05 |
| cg03776194 | 9.517404 | 3.142033 | 28.82878 | 6.76E-05 |
| cg10057098 | 1907.521 | 46.45296 | 78329.51 | 6.75E-05 |
| cg21495622 | 0.152617 | 0.060553 | 0.384654 | 6.73E-05 |
| cg03880841 | 0.001714 | 7.48E-05 | 0.039241 | 6.69E-05 |
| cg17947242 | 3640.324 | 64.64156 | 205006.8 | 6.69E-05 |
| cg27579097 | 0.086743 | 0.026078 | 0.288525 | 6.69E-05 |
| cg03578193 | 48.62279 | 7.210464 | 327.8812 | 6.64E-05 |
| cg13738652 | 0.170141 | 0.071261 | 0.406223 | 6.64E-05 |
| cg04045079 | 0.01781 | 0.002461 | 0.12889 | 6.64E-05 |
| cg14787477 | 0.136365 | 0.051254 | 0.362811 | 6.59E-05 |
| cg10654010 | 0.031148 | 0.00567 | 0.171107 | 6.57E-05 |
| cg06492521 | 0.24821 | 0.125249 | 0.491888 | 6.52E-05 |
| cg17419731 | 0.204518 | 0.093846 | 0.445705 | 6.52E-05 |
| cg09379601 | 11044.27 | 114.7074 | 1063365 | 6.47E-05 |
| cg21583690 | 4.91E+13 | 9427178 | 2.55E+20 | 6.46E-05 |
| cg21593554 | 68.45917 | 8.617696 | 543.8412 | 6.42E-05 |
| cg16015423 | 0.002308 | 0.000118 | 0.045254 | 6.37E-05 |
| cg13375571 | 0.249631 | 0.126457 | 0.492779 | 6.35E-05 |
| cg27508821 | 1.66E+08 | 15587.26 | 1.77E+12 | 6.33E-05 |
| cg26811705 | 0.134906 | 0.050567 | 0.359912 | 6.31E-05 |
| cg05944137 | 4.669568 | 2.195071 | 9.933557 | 6.3E-05 |
| cg03270881 | 0.003864 | 0.000254 | 0.058745 | 6.3E-05 |
| cg14417760 | 4.42524 | 2.135746 | 9.169045 | 6.3E-05 |
| cg19386379 | 0.039688 | 0.008172 | 0.192736 | 6.28E-05 |
| cg14989202 | 8.955621 | 3.060932 | 26.2022 | 6.27E-05 |
| cg14115756 | 0.256588 | 0.131808 | 0.499497 | 6.27E-05 |
| cg10095539 | 0.107882 | 0.036257 | 0.320995 | 6.27E-05 |
| cg24399337 | 0.028932 | 0.005108 | 0.163868 | 6.22E-05 |
| cg14186992 | 0.067504 | 0.018046 | 0.252508 | 6.21E-05 |
| cg02224369 | 0.030279 | 0.005468 | 0.167687 | 6.21E-05 |
| cg04400047 | 0.002494 | 0.000133 | 0.046844 | 6.19E-05 |
| cg12973017 | 0.06047 | 0.015323 | 0.238634 | 6.18E-05 |
| cg15925365 | 0.177133 | 0.075944 | 0.413149 | 6.18E-05 |
| cg08273772 | 0.004185 | 0.000287 | 0.061004 | 6.18E-05 |
| cg02632542 | 0.174243 | 0.074109 | 0.409675 | 6.18E-05 |
| cg22291359 | 0.039479 | 0.008125 | 0.191819 | 6.14E-05 |
| cg06319713 | 0.000755 | 2.25E-05 | 0.02534 | 6.07E-05 |
| cg01441777 | 0.07028 | 0.019213 | 0.257086 | 6E-05 |
| cg01419914 | 0.120332 | 0.04278 | 0.338471 | 5.99E-05 |
| cg27529346 | 7.457887 | 2.79613 | 19.89181 | 5.96E-05 |
| cg12359001 | 0.094576 | 0.029913 | 0.299021 | 5.93E-05 |
| cg24039042 | 7.001934 | 2.708272 | 18.10272 | 5.93E-05 |
| cg27520776 | 3.146027 | 1.79817 | 5.504199 | 5.92E-05 |
| cg24367850 | 0.071127 | 0.019583 | 0.258338 | 5.9E-05 |
| cg01157280 | 0.174878 | 0.074693 | 0.409442 | 5.89E-05 |
| cg24404823 | 0.267623 | 0.140677 | 0.509123 | 5.89E-05 |
| cg20600379 | 4.1987 | 2.085031 | 8.455068 | 5.89E-05 |
| cg17397004 | 0.153215 | 0.061359 | 0.382583 | 5.87E-05 |
| cg15528736 | 0.132219 | 0.049297 | 0.354618 | 5.83E-05 |
| cg04638014 | 0.237638 | 0.117957 | 0.478749 | 5.79E-05 |
| cg13505794 | 5.18E+08 | 29417.58 | 9.13E+12 | 5.75E-05 |
| cg12253175 | 0.179245 | 0.077575 | 0.414165 | 5.75E-05 |
| cg01504212 | 0.233257 | 0.114777 | 0.474039 | 5.74E-05 |
| cg13646917 | 0.050159 | 0.011675 | 0.215506 | 5.74E-05 |
| cg14606321 | 0.07042 | 0.019338 | 0.25643 | 5.73E-05 |
| cg11211259 | 0.003487 | 0.000222 | 0.054887 | 5.73E-05 |
| cg26348243 | 6.278288 | 2.566035 | 15.36101 | 5.72E-05 |
| cg25207828 | 0.05377 | 0.01295 | 0.223252 | 5.71E-05 |
| cg18729787 | 0.121273 | 0.043426 | 0.33867 | 5.67E-05 |
| cg02016178 | 7.986804 | 2.904756 | 21.9602 | 5.67E-05 |
| cg07313319 | 0.061871 | 0.015967 | 0.239743 | 5.66E-05 |
| cg22486834 | 0.151713 | 0.060592 | 0.379868 | 5.65E-05 |
| cg06358250 | 4.416399 | 2.143562 | 9.099147 | 5.64E-05 |
| cg24537986 | 0.030712 | 0.00564 | 0.167242 | 5.62E-05 |
| cg11397854 | 0.004354 | 0.000309 | 0.061337 | 5.62E-05 |
| cg23543481 | 0.100214 | 0.03272 | 0.306927 | 5.62E-05 |
| cg19701087 | 0.148514 | 0.058728 | 0.375569 | 5.61E-05 |
| cg01636582 | 0.022739 | 0.00361 | 0.143217 | 5.58E-05 |
| cg16362232 | 0.176468 | 0.075906 | 0.410258 | 5.58E-05 |
| cg01514075 | 0.185862 | 0.081993 | 0.421312 | 5.57E-05 |
| cg15852446 | 0.076669 | 0.021991 | 0.267293 | 5.56E-05 |
| cg21826978 | 36.21672 | 6.326599 | 207.3232 | 5.52E-05 |
| cg22507723 | 0.090658 | 0.028226 | 0.291186 | 5.52E-05 |
| cg13150534 | 0.125792 | 0.045932 | 0.344496 | 5.5E-05 |
| cg24453664 | 0.082542 | 0.024565 | 0.277357 | 5.49E-05 |
| cg02582774 | 10820185 | 4146.804 | 2.82E+10 | 5.45E-05 |
| cg27199523 | 1.16E+10 | 149824.7 | 8.93E+14 | 5.45E-05 |
| cg05992079 | 8.552182 | 3.015605 | 24.25378 | 5.45E-05 |
| cg00394658 | 0.116149 | 0.040832 | 0.330398 | 5.43E-05 |
| cg10115827 | 8.06E+08 | 38171.71 | 1.7E+13 | 5.43E-05 |
| cg12103475 | 0.022264 | 0.003511 | 0.141172 | 5.4E-05 |
| cg21642988 | 0.184022 | 0.080912 | 0.418528 | 5.4E-05 |
| cg06497051 | 0.040581 | 0.008566 | 0.192251 | 5.4E-05 |
| cg13460942 | 3.7E+20 | 3.85E+10 | 3.56E+30 | 5.39E-05 |
| cg17534029 | 0.174068 | 0.074513 | 0.406635 | 5.38E-05 |
| cg11287888 | 22.19048 | 4.933326 | 99.81444 | 5.34E-05 |
| cg01750654 | 0.199866 | 0.091568 | 0.436248 | 5.28E-05 |
| cg26034919 | 0.238782 | 0.119257 | 0.478101 | 5.27E-05 |
| cg25383568 | 72.65454 | 9.099622 | 580.0991 | 5.27E-05 |
| cg19015264 | 0.023065 | 0.003711 | 0.143373 | 5.27E-05 |
| cg16278747 | 0.128303 | 0.047438 | 0.347014 | 5.24E-05 |
| cg26406891 | 0.022375 | 0.003549 | 0.141057 | 5.24E-05 |
| cg24294399 | 9814.684 | 114.2504 | 843130.4 | 5.22E-05 |
| cg23364656 | 18.86726 | 4.546511 | 78.29598 | 5.22E-05 |
| cg23395310 | 0.026579 | 0.004586 | 0.15405 | 5.2E-05 |
| cg15689513 | 0.112827 | 0.039213 | 0.324634 | 5.2E-05 |
| cg02290550 | 0.111044 | 0.038301 | 0.321945 | 5.19E-05 |
| cg10277175 | 0.145297 | 0.057097 | 0.369743 | 5.17E-05 |
| cg12582959 | 0.073151 | 0.020621 | 0.259497 | 5.16E-05 |
| cg02608453 | 0.267095 | 0.140959 | 0.506103 | 5.16E-05 |
| cg05714559 | 0.189428 | 0.084669 | 0.423802 | 5.13E-05 |
| cg14269716 | 0.162916 | 0.067696 | 0.392068 | 5.13E-05 |
| cg19806182 | 0.139785 | 0.053958 | 0.362131 | 5.09E-05 |
| cg26672452 | 0.126932 | 0.046782 | 0.344397 | 5.05E-05 |
| cg05564831 | 0.076168 | 0.021939 | 0.264446 | 5.03E-05 |
| cg04969688 | 0.002966 | 0.000178 | 0.049444 | 5.02E-05 |
| cg03531211 | 17.29765 | 4.360713 | 68.61462 | 5.02E-05 |
| cg11511175 | 0.152147 | 0.06124 | 0.377999 | 5.01E-05 |
| cg18649823 | 0.000543 | 1.44E-05 | 0.020527 | 4.98E-05 |
| cg05694744 | 2.71E+08 | 22888.17 | 3.22E+12 | 4.96E-05 |
| cg10961323 | 0.241309 | 0.121446 | 0.47947 | 4.94E-05 |
| cg14282004 | 0.11329 | 0.039576 | 0.324303 | 4.94E-05 |
| cg19126910 | 9.148876 | 3.141577 | 26.64328 | 4.93E-05 |
| cg05361406 | 0.043847 | 0.009691 | 0.198379 | 4.9E-05 |
| cg21470947 | 5.399345 | 2.392424 | 12.18552 | 4.9E-05 |
| cg20570016 | 0.073191 | 0.020718 | 0.258568 | 4.9E-05 |
| cg25772438 | 0.007637 | 0.000726 | 0.080295 | 4.89E-05 |
| cg17618595 | 3.068814 | 1.786509 | 5.27152 | 4.86E-05 |
| cg14289461 | 0.128792 | 0.047909 | 0.346225 | 4.86E-05 |
| cg16363586 | 3.34013 | 1.867101 | 5.975291 | 4.82E-05 |
| cg04835284 | 0.179863 | 0.078639 | 0.411385 | 4.82E-05 |
| cg05037210 | 0.000395 | 9.04E-06 | 0.017298 | 4.81E-05 |
| cg25727093 | 0.039163 | 0.008213 | 0.186758 | 4.8E-05 |
| cg19577312 | 0.110277 | 0.038094 | 0.319238 | 4.8E-05 |
| cg18924331 | 0.179778 | 0.078603 | 0.411183 | 4.8E-05 |
| cg11800620 | 5.092252 | 2.323405 | 11.16079 | 4.79E-05 |
| cg03041617 | 0.207421 | 0.097183 | 0.442709 | 4.77E-05 |
| cg25577821 | 6.66898 | 2.672589 | 16.64128 | 4.76E-05 |
| cg27396498 | 5.793875 | 2.484759 | 13.50995 | 4.76E-05 |
| cg08343644 | 0.185271 | 0.082234 | 0.41741 | 4.74E-05 |
| cg17548735 | 0.050551 | 0.012009 | 0.212782 | 4.7E-05 |
| cg15600915 | 0.189445 | 0.085034 | 0.422055 | 4.69E-05 |
| cg26648948 | 0.032742 | 0.006312 | 0.169835 | 4.68E-05 |
| cg13829189 | 0.004992 | 0.000389 | 0.064021 | 4.67E-05 |
| cg01341487 | 0.063621 | 0.016891 | 0.239625 | 4.67E-05 |
| cg14173258 | 0.132006 | 0.049805 | 0.349877 | 4.67E-05 |
| cg02144874 | 0.161066 | 0.06689 | 0.387835 | 4.65E-05 |
| cg20102877 | 0.284354 | 0.155246 | 0.520832 | 4.65E-05 |
| cg16240137 | 555628.5 | 955.8101 | 3.23E+08 | 4.64E-05 |
| cg15379633 | 0.076511 | 0.022211 | 0.26356 | 4.64E-05 |
| cg24749947 | 0.012639 | 0.001543 | 0.103524 | 4.63E-05 |
| cg10142237 | 0.056708 | 0.014256 | 0.225567 | 4.62E-05 |
| cg15379170 | 0.075249 | 0.021683 | 0.261141 | 4.6E-05 |
| cg07497569 | 0.121982 | 0.044376 | 0.335305 | 4.54E-05 |
| cg08594606 | 0.235815 | 0.11778 | 0.472142 | 4.53E-05 |
| cg26897283 | 0.049574 | 0.011705 | 0.20995 | 4.51E-05 |
| cg21813538 | 0.089101 | 0.027891 | 0.284641 | 4.5E-05 |
| cg08550353 | 0.109106 | 0.037647 | 0.316204 | 4.49E-05 |
| cg22244122 | 0.024172 | 0.004045 | 0.144452 | 4.48E-05 |
| cg15817482 | 107.5843 | 11.37658 | 1017.387 | 4.48E-05 |
| cg03732535 | 0.007164 | 0.000669 | 0.076753 | 4.47E-05 |
| cg17187521 | 0.095022 | 0.030697 | 0.294141 | 4.45E-05 |
| cg25415932 | 0.02888 | 0.005268 | 0.15832 | 4.44E-05 |
| cg03562414 | 0.1538 | 0.062651 | 0.377557 | 4.39E-05 |
| cg16026647 | 0.090136 | 0.028418 | 0.285888 | 4.39E-05 |
| cg20804191 | 0.02638 | 0.004614 | 0.150814 | 4.38E-05 |
| cg07779444 | 0.024111 | 0.00404 | 0.143887 | 4.37E-05 |
| cg27500720 | 0.005537 | 0.000459 | 0.066844 | 4.34E-05 |
| cg14141458 | 0.005404 | 0.000443 | 0.065961 | 4.32E-05 |
| cg00798281 | 3.595252 | 1.947417 | 6.637426 | 4.3E-05 |
| cg00637477 | 0.132649 | 0.050393 | 0.349168 | 4.3E-05 |
| cg02774856 | 0.000107 | 1.34E-06 | 0.008551 | 4.3E-05 |
| cg22221320 | 3.257248 | 1.849981 | 5.735013 | 4.29E-05 |
| cg16823042 | 0.102803 | 0.034579 | 0.305636 | 4.27E-05 |
| cg22685502 | 0.103101 | 0.034743 | 0.305958 | 4.24E-05 |
| cg11650479 | 0.002932 | 0.00018 | 0.047828 | 4.23E-05 |
| cg03443944 | 0.075817 | 0.022056 | 0.260616 | 4.23E-05 |
| cg22324029 | 4.585525 | 2.212083 | 9.50554 | 4.23E-05 |
| cg08859278 | 8.681533 | 3.086484 | 24.41906 | 4.2E-05 |
| cg04966159 | 0.153046 | 0.06234 | 0.375733 | 4.2E-05 |
| cg03870261 | 0.152239 | 0.061856 | 0.374689 | 4.2E-05 |
| cg07058998 | 0.181542 | 0.080261 | 0.410631 | 4.18E-05 |
| cg10488031 | 0.046358 | 0.010668 | 0.20146 | 4.18E-05 |
| cg26458072 | 0.210668 | 0.100022 | 0.443713 | 4.17E-05 |
| cg00089550 | 0.093417 | 0.030078 | 0.290134 | 4.13E-05 |
| cg22820108 | 0.041989 | 0.009226 | 0.191089 | 4.12E-05 |
| cg06314111 | 0.073434 | 0.021086 | 0.255743 | 4.1E-05 |
| cg13857119 | 0.103079 | 0.034809 | 0.305247 | 4.09E-05 |
| cg08688548 | 0.174423 | 0.075732 | 0.401722 | 4.09E-05 |
| cg01987202 | 0.040356 | 0.00871 | 0.186988 | 4.07E-05 |
| cg02717339 | 0.182457 | 0.080972 | 0.411135 | 4.06E-05 |
| cg26309511 | 3.593721 | 1.951182 | 6.618978 | 4.04E-05 |
| cg22704780 | 0.104522 | 0.035564 | 0.307186 | 4.03E-05 |
| cg22621734 | 0.057255 | 0.014621 | 0.224211 | 4.01E-05 |
| cg22271905 | 0.020191 | 0.003135 | 0.130025 | 4.01E-05 |
| cg04907257 | 7.988647 | 2.963316 | 21.53617 | 4.01E-05 |
| cg06183267 | 4.934865 | 2.304059 | 10.56956 | 3.99E-05 |
| cg15375424 | 5.735845 | 2.492674 | 13.19864 | 3.99E-05 |
| cg10552523 | 5.150164 | 2.356392 | 11.25627 | 3.98E-05 |
| cg23902550 | 0.067188 | 0.01853 | 0.243626 | 3.98E-05 |
| cg06878361 | 0.107507 | 0.037106 | 0.311478 | 3.97E-05 |
| cg13307058 | 0.079204 | 0.023631 | 0.265473 | 3.97E-05 |
| cg22675956 | 0.010505 | 0.001196 | 0.092282 | 3.97E-05 |
| cg10999598 | 0.051099 | 0.012375 | 0.210994 | 3.95E-05 |
| cg02549492 | 0.034662 | 0.006977 | 0.172201 | 3.94E-05 |
| cg23545250 | 0.105468 | 0.036089 | 0.308225 | 3.94E-05 |
| cg16986624 | 126218.1 | 466.7479 | 34131938 | 3.94E-05 |
| cg08897388 | 0.020214 | 0.003148 | 0.129818 | 3.93E-05 |
| cg00119073 | 0.044609 | 0.010131 | 0.196411 | 3.92E-05 |
| cg02025583 | 0.151498 | 0.061626 | 0.372437 | 3.92E-05 |
| cg08402572 | 0.209243 | 0.099295 | 0.440931 | 3.9E-05 |
| cg07298473 | 0.090123 | 0.028634 | 0.283654 | 3.89E-05 |
| cg06991495 | 0.057848 | 0.014886 | 0.224809 | 3.87E-05 |
| cg00491548 | 0.069693 | 0.0196 | 0.247811 | 3.86E-05 |
| cg03241502 | 0.001616 | 7.57E-05 | 0.034472 | 3.84E-05 |
| cg15527643 | 0.054098 | 0.013492 | 0.216904 | 3.84E-05 |
| cg00549475 | 0.16035 | 0.067093 | 0.383231 | 3.83E-05 |
| cg00767000 | 0.310815 | 0.178217 | 0.542067 | 3.82E-05 |
| cg16045459 | 0.244986 | 0.125439 | 0.478467 | 3.82E-05 |
| cg11719297 | 0.009164 | 0.000983 | 0.085468 | 3.81E-05 |
| cg17792192 | 0.359321 | 0.22085 | 0.584611 | 3.76E-05 |
| cg06477487 | 0.12931 | 0.048892 | 0.341999 | 3.75E-05 |
| cg11453837 | 10.52711 | 3.437413 | 32.23938 | 3.75E-05 |
| cg08856772 | 907740.8 | 1334.392 | 6.18E+08 | 3.75E-05 |
| cg04286377 | 11.19883 | 3.551711 | 35.31082 | 3.74E-05 |
| cg07773434 | 0.127441 | 0.047872 | 0.339265 | 3.73E-05 |
| cg10535845 | 0.166869 | 0.071292 | 0.39058 | 3.68E-05 |
| cg00130180 | 0.132501 | 0.05074 | 0.346007 | 3.67E-05 |
| cg01195214 | 0.08076 | 0.024452 | 0.266737 | 3.66E-05 |
| cg14353649 | 0.058985 | 0.015387 | 0.226118 | 3.65E-05 |
| cg01607369 | 0.077959 | 0.023223 | 0.261701 | 3.63E-05 |
| cg21756647 | 0.15006 | 0.061007 | 0.369104 | 3.62E-05 |
| cg20702913 | 0.165557 | 0.070529 | 0.388619 | 3.61E-05 |
| cg13393830 | 0.207451 | 0.098363 | 0.437521 | 3.61E-05 |
| cg20655350 | 0.117432 | 0.042515 | 0.324362 | 3.6E-05 |
| cg12113740 | 0.187191 | 0.084563 | 0.414372 | 3.58E-05 |
| cg06017697 | 0.053681 | 0.013413 | 0.214844 | 3.58E-05 |
| cg08283130 | 0.118496 | 0.043104 | 0.325752 | 3.57E-05 |
| cg07570723 | 0.005359 | 0.000449 | 0.063939 | 3.57E-05 |
| cg17035591 | 13.33109 | 3.906055 | 45.49808 | 3.54E-05 |
| cg25446191 | 0.089117 | 0.028338 | 0.280256 | 3.53E-05 |
| cg14742148 | 0.00094 | 3.46E-05 | 0.02554 | 3.52E-05 |
| cg10512203 | 0.086719 | 0.027229 | 0.276185 | 3.52E-05 |
| cg26024851 | 0.001193 | 4.92E-05 | 0.028938 | 3.51E-05 |
| cg22533606 | 0.003392 | 0.00023 | 0.050144 | 3.5E-05 |
| cg27052073 | 0.127951 | 0.048331 | 0.338739 | 3.48E-05 |
| cg10661163 | 0.062761 | 0.01692 | 0.232797 | 3.48E-05 |
| cg11103390 | 0.119867 | 0.043904 | 0.327261 | 3.48E-05 |
| cg17905091 | 0.246314 | 0.126892 | 0.478127 | 3.47E-05 |
| cg08415592 | 4.434514 | 2.191046 | 8.975129 | 3.47E-05 |
| cg07607921 | 5.88E-08 | 2.22E-11 | 0.000156 | 3.46E-05 |
| cg23978657 | 3.333521 | 1.885346 | 5.894069 | 3.46E-05 |
| cg13802527 | 0.196957 | 0.091292 | 0.424925 | 3.45E-05 |
| cg24548564 | 3.742327 | 2.004032 | 6.988419 | 3.45E-05 |
| cg01971407 | 5.936849 | 2.555955 | 13.78983 | 3.44E-05 |
| cg18449389 | 0.021613 | 0.003523 | 0.132595 | 3.43E-05 |
| cg12513686 | 0.19905 | 0.092758 | 0.427142 | 3.42E-05 |
| cg09709457 | 0.182368 | 0.081586 | 0.407646 | 3.38E-05 |
| cg18964159 | 16970089 | 6495.925 | 4.43E+10 | 3.37E-05 |
| cg15418980 | 0.110431 | 0.03899 | 0.312771 | 3.35E-05 |
| cg03154077 | 28.63881 | 5.870953 | 139.7016 | 3.34E-05 |
| cg04880412 | 0.12291 | 0.045677 | 0.330733 | 3.31E-05 |
| cg06330621 | 0.062667 | 0.016953 | 0.231654 | 3.29E-05 |
| cg02924347 | 1.07E+17 | 9.85E+08 | 1.16E+25 | 3.28E-05 |
| cg05219593 | 0.009634 | 0.001077 | 0.086138 | 3.27E-05 |
| cg08690859 | 9.291914 | 3.248688 | 26.57678 | 3.22E-05 |
| cg05064489 | 0.164375 | 0.070178 | 0.385012 | 3.21E-05 |
| cg10337322 | 0.002106 | 0.000115 | 0.038429 | 3.19E-05 |
| cg26922780 | 17.08931 | 4.489911 | 65.04459 | 3.15E-05 |
| cg13296093 | 0.065274 | 0.018068 | 0.235815 | 3.12E-05 |
| cg14242936 | 0.065368 | 0.018107 | 0.235978 | 3.12E-05 |
| cg15700776 | 0.016698 | 0.002437 | 0.114403 | 3.07E-05 |
| cg20091689 | 0.213069 | 0.102991 | 0.440799 | 3.07E-05 |
| cg01613691 | 0.058037 | 0.015227 | 0.221212 | 3.05E-05 |
| cg21655969 | 0.004163 | 0.000317 | 0.054684 | 3.02E-05 |
| cg21869609 | 0.198588 | 0.092925 | 0.4244 | 3.02E-05 |
| cg09080173 | 0.026265 | 0.004753 | 0.145129 | 3.01E-05 |
| cg23964386 | 0.027988 | 0.005219 | 0.150088 | 3E-05 |
| cg09596958 | 0.16951 | 0.073654 | 0.390118 | 3E-05 |
| cg22923006 | 0.205799 | 0.097964 | 0.432337 | 2.99E-05 |
| cg05732300 | 0.004209 | 0.000323 | 0.054919 | 2.99E-05 |
| cg21939017 | 0.002155 | 0.000121 | 0.038488 | 2.98E-05 |
| cg13967702 | 6.080558 | 2.606241 | 14.1864 | 2.97E-05 |
| cg09809672 | 11.87005 | 3.717088 | 37.90552 | 2.96E-05 |
| cg13728106 | 0.218807 | 0.107238 | 0.44645 | 2.96E-05 |
| cg17327990 | 2.19E-06 | 4.84E-09 | 0.000992 | 2.96E-05 |
| cg06728055 | 0.038247 | 0.008273 | 0.176828 | 2.94E-05 |
| cg27320518 | 5.462056 | 2.462917 | 12.1133 | 2.94E-05 |
| cg16495212 | 0.114387 | 0.041369 | 0.316287 | 2.94E-05 |
| cg11063729 | 0.05938 | 0.015797 | 0.223201 | 2.92E-05 |
| cg13950558 | 0.139856 | 0.055609 | 0.351737 | 2.91E-05 |
| cg12894126 | 0.008247 | 0.00087 | 0.078186 | 2.9E-05 |
| cg12589431 | 0.053874 | 0.013702 | 0.211827 | 2.89E-05 |
| cg02343254 | 0.07618 | 0.022793 | 0.254611 | 2.89E-05 |
| cg27618939 | 0.034421 | 0.007098 | 0.166914 | 2.88E-05 |
| cg13718729 | 0.064533 | 0.017868 | 0.233075 | 2.88E-05 |
| cg16922937 | 0.282735 | 0.156427 | 0.511032 | 2.88E-05 |
| cg01220257 | 0.205721 | 0.09815 | 0.43119 | 2.82E-05 |
| cg08696931 | 0.034988 | 0.007292 | 0.167887 | 2.79E-05 |
| cg21174750 | 0.172146 | 0.075596 | 0.39201 | 2.79E-05 |
| cg08335254 | 0.073642 | 0.02174 | 0.249458 | 2.78E-05 |
| cg04895975 | 0.115566 | 0.042131 | 0.317001 | 2.77E-05 |
| cg07349094 | 7.933287 | 3.01548 | 20.87131 | 2.71E-05 |
| cg07777703 | 0.000157 | 2.64E-06 | 0.009391 | 2.7E-05 |
| cg05454446 | 0.039665 | 0.008794 | 0.178912 | 2.68E-05 |
| cg06850312 | 2.57E+14 | 48633968 | 1.36E+21 | 2.66E-05 |
| cg12487088 | 0.043256 | 0.009995 | 0.187193 | 2.65E-05 |
| cg14144201 | 0.025384 | 0.004575 | 0.140826 | 2.64E-05 |
| cg04020309 | 0.203642 | 0.096952 | 0.42774 | 2.64E-05 |
| cg07621169 | 0.199994 | 0.094434 | 0.423552 | 2.62E-05 |
| cg02806715 | 3.861592 | 2.057049 | 7.249167 | 2.62E-05 |
| cg15078329 | 0.023152 | 0.004002 | 0.133928 | 2.61E-05 |
| cg05708497 | 0.073394 | 0.021728 | 0.247918 | 2.6E-05 |
| cg17317391 | 0.171178 | 0.075246 | 0.389418 | 2.57E-05 |
| cg15825417 | 0.071198 | 0.020802 | 0.243684 | 2.56E-05 |
| cg16386697 | 0.138855 | 0.055389 | 0.3481 | 2.55E-05 |
| cg12135769 | 0.159437 | 0.067872 | 0.374535 | 2.51E-05 |
| cg16019612 | 0.109491 | 0.03915 | 0.306213 | 2.49E-05 |
| cg09438457 | 0.059614 | 0.016073 | 0.221111 | 2.48E-05 |
| cg14896463 | 0.028315 | 0.005404 | 0.148374 | 2.47E-05 |
| cg12504148 | 0.1504 | 0.062363 | 0.362718 | 2.47E-05 |
| cg20322611 | 0.008431 | 0.000917 | 0.077552 | 2.46E-05 |
| cg18702012 | 0.088446 | 0.028663 | 0.272913 | 2.46E-05 |
| cg20795913 | 0.072501 | 0.021437 | 0.245198 | 2.43E-05 |
| cg13009608 | 3.326071 | 1.904391 | 5.809075 | 2.4E-05 |
| cg25221637 | 0.118522 | 0.044061 | 0.318823 | 2.4E-05 |
| cg14318734 | 0.015509 | 0.002244 | 0.107178 | 2.4E-05 |
| cg08310837 | 0.085305 | 0.027229 | 0.267251 | 2.39E-05 |
| cg09214398 | 0.260879 | 0.139868 | 0.486589 | 2.39E-05 |
| cg13253439 | 0.044833 | 0.010622 | 0.189226 | 2.38E-05 |
| cg09028651 | 0.059035 | 0.015895 | 0.219256 | 2.37E-05 |
| cg12041075 | 0.098566 | 0.033667 | 0.28857 | 2.36E-05 |
| cg13506354 | 0.186278 | 0.085472 | 0.405973 | 2.36E-05 |
| cg15937073 | 0.039661 | 0.008884 | 0.177063 | 2.36E-05 |
| cg19878482 | 0.119545 | 0.04466 | 0.32 | 2.36E-05 |
| cg00619978 | 0.018801 | 0.002981 | 0.118563 | 2.34E-05 |
| cg20366832 | 0.113796 | 0.041571 | 0.311505 | 2.34E-05 |
| cg08321366 | 0.022514 | 0.003882 | 0.130555 | 2.33E-05 |
| cg21826784 | 0.067202 | 0.019237 | 0.234754 | 2.33E-05 |
| cg07340894 | 0.037521 | 0.008208 | 0.171517 | 2.3E-05 |
| cg22120446 | 14.00955 | 4.128203 | 47.54306 | 2.29E-05 |
| cg04211275 | 6031.908 | 107.4613 | 338576.8 | 2.28E-05 |
| cg03505148 | 0.10286 | 0.035911 | 0.294626 | 2.28E-05 |
| cg09692695 | 0.09959 | 0.034258 | 0.289519 | 2.27E-05 |
| cg13784235 | 8.768641 | 3.212342 | 23.93552 | 2.26E-05 |
| cg10829227 | 0.119418 | 0.044702 | 0.319016 | 2.25E-05 |
| cg21182407 | 0.000392 | 1.04E-05 | 0.014734 | 2.24E-05 |
| cg05931439 | 0.060791 | 0.016658 | 0.221853 | 2.24E-05 |
| cg03835987 | 0.130677 | 0.051006 | 0.334792 | 2.24E-05 |
| cg19374305 | 0.08779 | 0.028511 | 0.270324 | 2.24E-05 |
| cg07013258 | 0.012485 | 0.001648 | 0.094583 | 2.21E-05 |
| cg27313492 | 0.074523 | 0.022463 | 0.247234 | 2.2E-05 |
| cg03984055 | 0.047073 | 0.011478 | 0.193051 | 2.19E-05 |
| cg07047068 | 0.071272 | 0.021061 | 0.241186 | 2.17E-05 |
| cg05937445 | 0.09521 | 0.032161 | 0.281862 | 2.17E-05 |
| cg01912298 | 0.251809 | 0.133252 | 0.475849 | 2.17E-05 |
| cg23679769 | 0.000434 | 1.22E-05 | 0.015382 | 2.11E-05 |
| cg21936280 | 0.011441 | 0.00146 | 0.089678 | 2.09E-05 |
| cg11817892 | 0.2139 | 0.105158 | 0.435093 | 2.07E-05 |
| cg11613875 | 65009.25 | 396.1439 | 10668354 | 2.06E-05 |
| cg14585353 | 54.9517 | 8.693179 | 347.3631 | 2.06E-05 |
| cg19018267 | 0.038577 | 0.00863 | 0.172446 | 2.04E-05 |
| cg17330838 | 0.123695 | 0.047298 | 0.323494 | 2.04E-05 |
| cg11639950 | 0.094756 | 0.032064 | 0.28002 | 2.02E-05 |
| cg19729479 | 55.73933 | 8.777052 | 353.9768 | 2.02E-05 |
| cg16284674 | 0.040524 | 0.009285 | 0.176855 | 2E-05 |
| cg26999345 | 0.038181 | 0.008527 | 0.17096 | 1.96E-05 |
| cg21485555 | 7110.604 | 121.294 | 416844.3 | 1.95E-05 |
| cg11523350 | 0.203395 | 0.097952 | 0.422346 | 1.94E-05 |
| cg24713204 | 0.069695 | 0.020546 | 0.236416 | 1.92E-05 |
| cg06707910 | 0.029159 | 0.005767 | 0.147423 | 1.91E-05 |
| cg26588076 | 0.114344 | 0.04233 | 0.308872 | 1.89E-05 |
| cg17720013 | 0.194998 | 0.092209 | 0.412371 | 1.88E-05 |
| cg07871024 | 0.131302 | 0.051801 | 0.332816 | 1.88E-05 |
| cg11596009 | 0.065229 | 0.018682 | 0.227754 | 1.88E-05 |
| cg15128365 | 0.137561 | 0.055479 | 0.34108 | 1.86E-05 |
| cg19482025 | 0.100798 | 0.035274 | 0.288041 | 1.84E-05 |
| cg05905531 | 0.038269 | 0.008601 | 0.170285 | 1.83E-05 |
| cg06885782 | 0.125559 | 0.04862 | 0.32425 | 1.81E-05 |
| cg01081346 | 0.209229 | 0.102362 | 0.427664 | 1.8E-05 |
| cg12432010 | 0.011239 | 0.001446 | 0.087367 | 1.79E-05 |
| cg15784615 | 0.029661 | 0.005947 | 0.147922 | 1.78E-05 |
| cg24766690 | 0.020109 | 0.003379 | 0.119669 | 1.76E-05 |
| cg04833514 | 0.105 | 0.037531 | 0.29376 | 1.76E-05 |
| cg08583411 | 0.069732 | 0.020698 | 0.234936 | 1.73E-05 |
| cg18587504 | 0.007276 | 0.000771 | 0.068698 | 1.73E-05 |
| cg18892212 | 0.144591 | 0.059862 | 0.349249 | 1.72E-05 |
| cg05685023 | 0.042343 | 0.010015 | 0.179018 | 1.72E-05 |
| cg23060272 | 0.006851 | 0.000706 | 0.066452 | 1.72E-05 |
| cg04357965 | 0.121807 | 0.046645 | 0.318087 | 1.72E-05 |
| cg15999590 | 0.04213 | 0.009946 | 0.178449 | 1.71E-05 |
| cg09635667 | 0.051608 | 0.013367 | 0.199248 | 1.7E-05 |
| cg15340334 | 51.1 | 8.507815 | 306.919 | 1.7E-05 |
| cg22033586 | 0.073789 | 0.022504 | 0.241947 | 1.69E-05 |
| cg08100069 | 0.110655 | 0.040598 | 0.301602 | 1.69E-05 |
| cg07664173 | 0.000721 | 2.68E-05 | 0.019432 | 1.67E-05 |
| cg04999352 | 4.763458 | 2.340739 | 9.693746 | 1.66E-05 |
| cg07551364 | 0.040903 | 0.00955 | 0.175197 | 1.66E-05 |
| cg09730719 | 0.045855 | 0.011278 | 0.186439 | 1.65E-05 |
| cg02576938 | 0.151448 | 0.064169 | 0.357436 | 1.65E-05 |
| cg17526952 | 218.068 | 18.8608 | 2521.295 | 1.62E-05 |
| cg21551549 | 0.040442 | 0.009421 | 0.173595 | 1.59E-05 |
| cg03052606 | 0.005417 | 0.000507 | 0.057912 | 1.59E-05 |
| cg26336059 | 0.023936 | 0.004398 | 0.130272 | 1.58E-05 |
| cg26418434 | 0.118492 | 0.045013 | 0.311916 | 1.57E-05 |
| cg04993257 | 0.059036 | 0.01635 | 0.213165 | 1.56E-05 |
| cg04393837 | 0.114323 | 0.042737 | 0.305814 | 1.56E-05 |
| cg08450982 | 0.128857 | 0.050865 | 0.326439 | 1.56E-05 |
| cg24630419 | 6.194426 | 2.70886 | 14.16496 | 1.55E-05 |
| cg03852570 | 0.034388 | 0.007458 | 0.158563 | 1.55E-05 |
| cg08055330 | 0.154092 | 0.066041 | 0.359538 | 1.52E-05 |
| cg02285386 | 0.201406 | 0.09746 | 0.416212 | 1.51E-05 |
| cg23691642 | 0.074996 | 0.023205 | 0.242377 | 1.51E-05 |
| cg10615591 | 0.159854 | 0.069697 | 0.366638 | 1.5E-05 |
| cg15043384 | 0.084469 | 0.027597 | 0.258546 | 1.49E-05 |
| cg14218480 | 0.121451 | 0.046778 | 0.315328 | 1.49E-05 |
| cg03000846 | 0.136994 | 0.055769 | 0.336521 | 1.46E-05 |
| cg15409237 | 0.161311 | 0.070722 | 0.367936 | 1.45E-05 |
| cg14967066 | 3.927339 | 2.11724 | 7.28495 | 1.43E-05 |
| cg02482460 | 3.398031 | 1.955784 | 5.90383 | 1.43E-05 |
| cg10975001 | 0.062941 | 0.018059 | 0.219376 | 1.42E-05 |
| cg13868216 | 0.118763 | 0.045394 | 0.310713 | 1.41E-05 |
| cg25004071 | 0.147823 | 0.062424 | 0.350052 | 1.38E-05 |
| cg10643916 | 0.041306 | 0.009817 | 0.1738 | 1.38E-05 |
| cg26253974 | 0.008101 | 0.000924 | 0.071051 | 1.38E-05 |
| cg01248010 | 2.69E-06 | 8.28E-09 | 0.000873 | 1.38E-05 |
| cg00933835 | 0.10155 | 0.036222 | 0.284702 | 1.37E-05 |
| cg13832201 | 0.060813 | 0.017219 | 0.214769 | 1.37E-05 |
| cg23093496 | 10.67132 | 3.672401 | 31.00889 | 1.36E-05 |
| cg19657875 | 2.3E-06 | 6.65E-09 | 0.000794 | 1.34E-05 |
| cg21365602 | 3.132382 | 1.873639 | 5.236771 | 1.33E-05 |
| cg23095383 | 67.49922 | 10.13847 | 449.3918 | 1.33E-05 |
| cg07747241 | 2559705 | 3348.851 | 1.96E+09 | 1.32E-05 |
| cg09572067 | 0.068249 | 0.020408 | 0.228248 | 1.31E-05 |
| cg02865595 | 0.12609 | 0.049695 | 0.319929 | 1.31E-05 |
| cg24312537 | 0.159235 | 0.069743 | 0.363561 | 1.29E-05 |
| cg22437987 | 0.020757 | 0.00364 | 0.118371 | 1.29E-05 |
| cg25635864 | 0.024736 | 0.004695 | 0.130336 | 1.28E-05 |
| cg11763337 | 453.5441 | 29.07119 | 7075.81 | 1.28E-05 |
| cg01328833 | 0.086867 | 0.02901 | 0.260109 | 1.26E-05 |
| cg04177132 | 0.013991 | 0.002059 | 0.095065 | 1.26E-05 |
| cg05210373 | 0.135646 | 0.05534 | 0.332487 | 1.26E-05 |
| cg01465361 | 2790412 | 3572.239 | 2.18E+09 | 1.26E-05 |
| cg13772815 | 0.102336 | 0.036805 | 0.284544 | 1.25E-05 |
| cg22306579 | 0.04504 | 0.011217 | 0.180845 | 1.24E-05 |
| cg00430484 | 0.094419 | 0.032781 | 0.271952 | 1.23E-05 |
| cg11797228 | 5921515 | 5504.85 | 6.37E+09 | 1.2E-05 |
| cg26151910 | 148.3864 | 15.82768 | 1391.14 | 1.19E-05 |
| cg15095913 | 0.05996 | 0.01702 | 0.211236 | 1.19E-05 |
| cg00835193 | 0.143763 | 0.060394 | 0.342212 | 1.17E-05 |
| cg05733780 | 7.536409 | 3.055073 | 18.59119 | 1.16E-05 |
| cg26741686 | 0.21407 | 0.107482 | 0.426359 | 1.16E-05 |
| cg08397758 | 0.066335 | 0.019772 | 0.222554 | 1.12E-05 |
| cg17393635 | 0.106645 | 0.039312 | 0.289307 | 1.1E-05 |
| cg21917349 | 19.98893 | 5.259055 | 75.9751 | 1.1E-05 |
| cg20801110 | 0.15597 | 0.068126 | 0.357082 | 1.1E-05 |
| cg02737321 | 0.005108 | 0.000487 | 0.053629 | 1.09E-05 |
| cg13938098 | 0.067635 | 0.020377 | 0.224491 | 1.08E-05 |
| cg18909903 | 0.087358 | 0.029499 | 0.258698 | 1.08E-05 |
| cg23120601 | 0.145648 | 0.061768 | 0.343431 | 1.07E-05 |
| cg16280132 | 10.46598 | 3.679479 | 29.76962 | 1.07E-05 |
| cg17656260 | 0.000179 | 3.85E-06 | 0.008338 | 1.07E-05 |
| cg16787352 | 0.122414 | 0.04807 | 0.311737 | 1.06E-05 |
| cg14947634 | 0.249368 | 0.134478 | 0.462412 | 1.04E-05 |
| cg19190163 | 9.234583 | 3.438586 | 24.80017 | 1.03E-05 |
| cg03223659 | 1009.835 | 46.81177 | 21784.4 | 1.01E-05 |
| cg00784882 | 0.105229 | 0.038757 | 0.285707 | 9.95E-06 |
| cg17954373 | 0.000161 | 3.36E-06 | 0.00776 | 9.9E-06 |
| cg01468220 | 0.198334 | 0.096811 | 0.406318 | 9.81E-06 |
| cg01024247 | 0.09219 | 0.032074 | 0.264987 | 9.63E-06 |
| cg02335376 | 0.053027 | 0.014442 | 0.194702 | 9.62E-06 |
| cg21806242 | 0.105292 | 0.038864 | 0.285259 | 9.57E-06 |
| cg22027399 | 0.058547 | 0.016665 | 0.205682 | 9.57E-06 |
| cg19619576 | 0.187305 | 0.089254 | 0.39307 | 9.47E-06 |
| cg08877374 | 0.06963 | 0.021421 | 0.226337 | 9.41E-06 |
| cg19887750 | 0.023243 | 0.004405 | 0.122643 | 9.3E-06 |
| cg11658419 | 0.07383 | 0.023332 | 0.233615 | 9.25E-06 |
| cg23923934 | 7.713857 | 3.132575 | 18.9951 | 8.85E-06 |
| cg04563438 | 0.002674 | 0.000196 | 0.036413 | 8.73E-06 |
| cg10599444 | 0.054885 | 0.015269 | 0.19728 | 8.73E-06 |
| cg18359371 | 0.042572 | 0.010597 | 0.171022 | 8.63E-06 |
| cg09514174 | 0.048022 | 0.01261 | 0.182878 | 8.58E-06 |
| cg07538190 | 0.071191 | 0.022243 | 0.227852 | 8.51E-06 |
| cg17079034 | 0.019487 | 0.003443 | 0.110291 | 8.48E-06 |
| cg10817441 | 5.514878 | 2.600995 | 11.69317 | 8.48E-06 |
| cg00851518 | 0.112183 | 0.042853 | 0.29368 | 8.38E-06 |
| cg13067908 | 0.005106 | 0.000502 | 0.051948 | 8.25E-06 |
| cg27518898 | 0.070158 | 0.021824 | 0.225543 | 8.22E-06 |
| cg13095627 | 0.243583 | 0.130944 | 0.453118 | 8.21E-06 |
| cg00244517 | 0.182855 | 0.086724 | 0.385543 | 8.04E-06 |
| cg00610692 | 0.03652 | 0.008541 | 0.156154 | 8.01E-06 |
| cg12748890 | 0.140899 | 0.059628 | 0.332937 | 7.94E-06 |
| cg05564251 | 17.73526 | 5.024384 | 62.60258 | 7.87E-06 |
| cg16359550 | 5.839821 | 2.69354 | 12.66123 | 7.84E-06 |
| cg11187245 | 6.56006 | 2.87683 | 14.95896 | 7.73E-06 |
| cg24898914 | 13.4922 | 4.314662 | 42.1909 | 7.7E-06 |
| cg22595230 | 0.131686 | 0.054194 | 0.319984 | 7.63E-06 |
| cg18351607 | 0.002894 | 0.000224 | 0.037378 | 7.55E-06 |
| cg07343703 | 0.186619 | 0.08951 | 0.389082 | 7.53E-06 |
| cg23725321 | 0.170547 | 0.078661 | 0.369768 | 7.48E-06 |
| cg07543823 | 0.022771 | 0.004357 | 0.119007 | 7.37E-06 |
| cg14719333 | 6.706668 | 2.918691 | 15.41081 | 7.35E-06 |
| cg08345719 | 0.086958 | 0.029914 | 0.252784 | 7.26E-06 |
| cg14591340 | 0.146328 | 0.063203 | 0.338783 | 7.22E-06 |
| cg06759518 | 0.057009 | 0.016347 | 0.198814 | 6.97E-06 |
| cg07839457 | 4.511921 | 2.339178 | 8.702814 | 6.94E-06 |
| cg05948411 | 0.052893 | 0.01469 | 0.190454 | 6.89E-06 |
| cg14413165 | 0.027513 | 0.005748 | 0.131702 | 6.88E-06 |
| cg26136365 | 0.029359 | 0.006317 | 0.136462 | 6.77E-06 |
| cg13761843 | 0.040022 | 0.009863 | 0.162402 | 6.68E-06 |
| cg02792829 | 0.047007 | 0.012426 | 0.177823 | 6.67E-06 |
| cg03950476 | 0.098695 | 0.036032 | 0.270335 | 6.66E-06 |
| cg21184369 | 0.231858 | 0.122851 | 0.437587 | 6.47E-06 |
| cg19300923 | 0.114383 | 0.044601 | 0.293344 | 6.41E-06 |
| cg04545296 | 0.016522 | 0.002791 | 0.097806 | 6.12E-06 |
| cg08469215 | 0.113027 | 0.04394 | 0.290736 | 6.11E-06 |
| cg05783384 | 0.109592 | 0.042109 | 0.285223 | 5.88E-06 |
| cg14091103 | 0.093245 | 0.033428 | 0.260101 | 5.82E-06 |
| cg18030218 | 8.97E+30 | 3.75E+17 | 2.15E+44 | 5.77E-06 |
| cg03520342 | 4.768719 | 2.427648 | 9.367374 | 5.77E-06 |
| cg11058730 | 5.81E+08 | 95729.22 | 3.53E+12 | 5.61E-06 |
| cg25141995 | 0.129182 | 0.053447 | 0.312234 | 5.49E-06 |
| cg11801411 | 0.100249 | 0.03719 | 0.270227 | 5.46E-06 |
| cg08157684 | 0.006045 | 0.00067 | 0.054556 | 5.33E-06 |
| cg00730561 | 0.192184 | 0.094542 | 0.39067 | 5.2E-06 |
| cg17453840 | 0.191851 | 0.094322 | 0.390227 | 5.17E-06 |
| cg03753191 | 5.400898 | 2.616399 | 11.1488 | 5.09E-06 |
| cg15736127 | 0.075173 | 0.024723 | 0.228575 | 5.09E-06 |
| cg09233429 | 0.119992 | 0.048253 | 0.298385 | 5.07E-06 |
| cg04247135 | 0.08181 | 0.027926 | 0.239668 | 5E-06 |
| cg16393207 | 0.179358 | 0.085776 | 0.375041 | 4.98E-06 |
| cg19966212 | 3.126613 | 1.917921 | 5.097035 | 4.84E-06 |
| cg12532266 | 0.15085 | 0.0671 | 0.339133 | 4.73E-06 |
| cg03339910 | 0.084522 | 0.029352 | 0.243386 | 4.68E-06 |
| cg24179027 | 0.087955 | 0.031085 | 0.248873 | 4.63E-06 |
| cg15719903 | 15.34116 | 4.770707 | 49.33254 | 4.61E-06 |
| cg14856698 | 0.002263 | 0.000167 | 0.030585 | 4.55E-06 |
| cg15299835 | 0.019519 | 0.003629 | 0.104975 | 4.52E-06 |
| cg05666287 | 0.127564 | 0.052923 | 0.307473 | 4.49E-06 |
| cg08159663 | 7.86122 | 3.258917 | 18.96299 | 4.44E-06 |
| cg02860797 | 0.012525 | 0.00193 | 0.081302 | 4.44E-06 |
| cg14625154 | 20.72757 | 5.685626 | 75.56461 | 4.36E-06 |
| cg12017631 | 0.116381 | 0.046487 | 0.291363 | 4.35E-06 |
| cg10114555 | 0.028456 | 0.006232 | 0.129928 | 4.35E-06 |
| cg06230674 | 0.029478 | 0.006558 | 0.132509 | 4.32E-06 |
| cg00754357 | 0.150574 | 0.067164 | 0.337567 | 4.3E-06 |
| cg14292823 | 0.056606 | 0.016662 | 0.192301 | 4.18E-06 |
| cg08100159 | 0.22144 | 0.116576 | 0.420635 | 4.12E-06 |
| cg21800196 | 0.14085 | 0.061168 | 0.324331 | 4.11E-06 |
| cg15537850 | 0.063512 | 0.019696 | 0.204796 | 3.94E-06 |
| cg23632849 | 0.050403 | 0.014186 | 0.179089 | 3.86E-06 |
| cg14131038 | 0.060233 | 0.018287 | 0.198392 | 3.85E-06 |
| cg16186435 | 8.43374 | 3.412968 | 20.8405 | 3.85E-06 |
| cg23359895 | 968518.6 | 2796.334 | 3.35E+08 | 3.84E-06 |
| cg04044188 | 0.165126 | 0.076958 | 0.354305 | 3.77E-06 |
| cg10369594 | 0.069061 | 0.022256 | 0.214291 | 3.72E-06 |
| cg09321817 | 4.040515 | 2.236939 | 7.298257 | 3.68E-06 |
| cg02374486 | 17.64589 | 5.233724 | 59.49441 | 3.67E-06 |
| cg25095994 | 5.302311 | 2.619324 | 10.7335 | 3.55E-06 |
| cg12258811 | 0.027148 | 0.005917 | 0.124565 | 3.49E-06 |
| cg23288103 | 0.077504 | 0.026311 | 0.228307 | 3.49E-06 |
| cg18869127 | 0.07171 | 0.023561 | 0.218257 | 3.48E-06 |
| cg25422089 | 0.159841 | 0.073714 | 0.346599 | 3.43E-06 |
| cg20321801 | 0.045759 | 0.012453 | 0.168137 | 3.4E-06 |
| cg06791592 | 8.423043 | 3.433931 | 20.66077 | 3.24E-06 |
| cg06414073 | 0.008686 | 0.001178 | 0.064027 | 3.21E-06 |
| cg25900902 | 0.176256 | 0.084923 | 0.365816 | 3.17E-06 |
| cg19028462 | 0.179457 | 0.087284 | 0.368963 | 2.99E-06 |
| cg22128918 | 0.035157 | 0.008642 | 0.143024 | 2.92E-06 |
| cg03750567 | 0.097754 | 0.036923 | 0.2588 | 2.85E-06 |
| cg21161394 | 0.064293 | 0.02044 | 0.202235 | 2.68E-06 |
| cg01768328 | 0.108969 | 0.043195 | 0.274895 | 2.66E-06 |
| cg15895213 | 0.001075 | 6.2E-05 | 0.018644 | 2.66E-06 |
| cg24129356 | 5.219845 | 2.619347 | 10.40213 | 2.64E-06 |
| cg01472113 | 0.146246 | 0.0656 | 0.326035 | 2.6E-06 |
| cg03331514 | 0.091512 | 0.033875 | 0.247215 | 2.4E-06 |
| cg20613400 | 0.004155 | 0.000426 | 0.040552 | 2.39E-06 |
| cg04803153 | 0.126517 | 0.05361 | 0.298572 | 2.37E-06 |
| cg24768595 | 0.220858 | 0.118152 | 0.412844 | 2.22E-06 |
| cg05087067 | 0.129955 | 0.055939 | 0.301902 | 2.09E-06 |
| cg02056062 | 0.023201 | 0.004907 | 0.109699 | 2.05E-06 |
| cg00666746 | 0.036088 | 0.00921 | 0.141414 | 1.87E-06 |
| cg26299084 | 0.046993 | 0.013392 | 0.164897 | 1.8E-06 |
| cg15818800 | 59.51117 | 11.12393 | 318.3747 | 1.79E-06 |
| cg00533183 | 7.982305 | 3.430071 | 18.57606 | 1.43E-06 |
| cg20831708 | 0.16409 | 0.078813 | 0.341639 | 1.36E-06 |
| cg18511546 | 18.73991 | 5.718857 | 61.40814 | 1.3E-06 |
| cg01502320 | 51.59103 | 10.45052 | 254.6891 | 1.3E-06 |
| cg02160608 | 0.053676 | 0.016428 | 0.175377 | 1.29E-06 |
| cg10536276 | 0.069863 | 0.023862 | 0.20455 | 1.2E-06 |
| cg25621286 | 46.74122 | 9.912732 | 220.3976 | 1.18E-06 |
| cg15442268 | 0.01334 | 0.002353 | 0.075639 | 1.08E-06 |
| cg14080585 | 0.078111 | 0.028085 | 0.217245 | 1.03E-06 |
| cg17186163 | 0.012904 | 0.002262 | 0.073611 | 9.75E-07 |
| cg14019050 | 0.102929 | 0.041512 | 0.255208 | 9.21E-07 |
| cg21163717 | 18.89078 | 5.842865 | 61.07647 | 9.19E-07 |
| cg14333539 | 0.097879 | 0.038757 | 0.247188 | 8.8E-07 |
| cg11863058 | 0.169237 | 0.0835 | 0.343005 | 8.28E-07 |
| cg21548131 | 0.160887 | 0.0779 | 0.332281 | 7.92E-07 |
| cg00826767 | 0.140561 | 0.064688 | 0.305423 | 7.22E-07 |
| cg22493877 | 0.019964 | 0.0043 | 0.092696 | 5.85E-07 |
| cg23925513 | 0.047565 | 0.014421 | 0.15689 | 5.68E-07 |
| cg00622799 | 0.054415 | 0.017418 | 0.169997 | 5.48E-07 |
| cg14204433 | 0.057348 | 0.01882 | 0.17475 | 4.95E-07 |
| cg16622899 | 0.037465 | 0.010462 | 0.134158 | 4.5E-07 |
| cg09468328 | 0.020465 | 0.004519 | 0.092678 | 4.5E-07 |
| cg27048142 | 0.145135 | 0.068914 | 0.305659 | 3.79E-07 |
| cg18489607 | 0.045515 | 0.013845 | 0.149634 | 3.61E-07 |
| cg27285720 | 4.235343 | 2.432789 | 7.373482 | 3.35E-07 |
| cg26099902 | 0.130644 | 0.059873 | 0.285071 | 3.18E-07 |
| cg05949660 | 0.065971 | 0.023277 | 0.186978 | 3.14E-07 |
| cg07808761 | 0.078676 | 0.029858 | 0.207311 | 2.7E-07 |
| cg04661436 | 0.137506 | 0.06463 | 0.292554 | 2.59E-07 |
| cg11274940 | 0.035515 | 0.009985 | 0.12632 | 2.53E-07 |
| cg07005444 | 0.030747 | 0.008261 | 0.114442 | 2.07E-07 |
| cg17209284 | 0.135566 | 0.064562 | 0.284658 | 1.29E-07 |
| cg07156249 | 5.106738 | 2.789133 | 9.350137 | 1.26E-07 |
| cg16449084 | 0.0686 | 0.02569 | 0.183183 | 8.95E-08 |
| cg02736280 | 0.106352 | 0.048106 | 0.235121 | 3.09E-08 |
| cg21824343 | 0.09304 | 0.040286 | 0.214872 | 2.69E-08 |
| cg23152216 | 0.071195 | 0.028313 | 0.179026 | 1.95E-08 |
| cg13206063 | 0.080213 | 0.033984 | 0.189323 | 8.49E-09 |
