# Supplementary material for: Identification of Melanoma Subsets Based on DNA Methylation Sites and Construction of a Prognosis Evaluation Model
Source: J Oncol. 2022 Oct 11;2022:6608650. doi: 10.1155/2022/6608650 (PMC9578801; doi:10.1155/2022/6608650)
Supplement: Supplementary Materials — Supplementary Table 1. Prognosis-related methylation sites by univariate Cox regression analysis (783 sites were found, P < 0.0001). Supplementary Table 2. Multivariate Cox regression analysis of the 783 methylation sites (256 sites were found, P < 0.0001). Supplementary Table 3. Level of the 256 sites in 338 samples and the follow-up. Supplementary Table 4. Analysis of differences in methylation site levels between the 7 clusters. Supplementary Table 5. Risk assessment. Supplementary Table 6. Testing the prediction model in 60% of the samples (randomly) for 100 times. Supplementary Table 7. Functional enrichment analysis of genes and 28 pathways were observed. Supplementary Table 8. The correlation coefficients between the expression levels of the 35 hub genes (∗∗P < 0.01, ∗P < 0.05). Supplementary Table 9. The correlation coefficients of the critical genes validated in the testing group (∗∗P < 0.01). Figure S1. The clinical features in different DNA methylation subgroups. The clinical T categories (a), N stage (b), and M status (c) of different subtypes. The tumor stage (d), patient ages (e), and gender (f) in different subtypes. C, cluster; T, primary tumor; N, lymph node involvement; M, distant metastases. [file 6608650.f1.zip › Supplementary Table 2. Multivariate Cox regression analysis of the 783 methylation sites.docx]

| **Supplementary Table 2. Multivariate Cox regression analysis of the 783 methylation sites (256 sites were found, *P*<0.0001).** | | | | |
| --- | --- | --- | --- | --- |
| **ID** | **HR** | **Low.95%CI** | **High.95%CI** | **p-value** |
| cg00089550 | 0.072005 | 0.02337 | 0.221855 | 4.59E-06 |
| cg00119073 | 0.032497 | 0.007221 | 0.14624 | 8.00E-06 |
| cg00244517 | 0.168896 | 0.079753 | 0.35768 | 3.39E-06 |
| cg00394658 | 0.071381 | 0.025025 | 0.203601 | 7.97E-07 |
| cg00491548 | 0.057392 | 0.015872 | 0.207525 | 1.31E-05 |
| cg00533183 | 7.268384 | 2.974397 | 17.76139 | 1.35E-05 |
| cg00549475 | 0.123717 | 0.050947 | 0.300426 | 3.90E-06 |
| cg00622799 | 0.057277 | 0.018332 | 0.17896 | 8.65E-07 |
| cg00637477 | 0.09902 | 0.038116 | 0.257242 | 2.06E-06 |
| cg00666746 | 0.028971 | 0.007301 | 0.114958 | 4.75E-07 |
| cg00730561 | 0.185941 | 0.089531 | 0.386171 | 6.43E-06 |
| cg00754357 | 0.134472 | 0.058619 | 0.30848 | 2.18E-06 |
| cg00835193 | 0.135985 | 0.05538 | 0.333908 | 1.34E-05 |
| cg00851518 | 0.119392 | 0.046217 | 0.308423 | 1.14E-05 |
| cg00933835 | 0.106234 | 0.038094 | 0.296258 | 1.83E-05 |
| cg01024247 | 0.085599 | 0.029683 | 0.246849 | 5.39E-06 |
| cg01220257 | 0.191501 | 0.090132 | 0.406877 | 1.72E-05 |
| cg01328833 | 0.068513 | 0.022404 | 0.20952 | 2.60E-06 |
| cg01341487 | 0.048786 | 0.012601 | 0.188883 | 1.23E-05 |
| cg01613691 | 0.055761 | 0.015178 | 0.204858 | 1.37E-05 |
| cg01768328 | 0.11192 | 0.043551 | 0.287617 | 5.43E-06 |
| cg02025583 | 0.117116 | 0.047248 | 0.290304 | 3.65E-06 |
| cg02055253 | 0.090189 | 0.034478 | 0.235921 | 9.40E-07 |
| cg02160608 | 0.061608 | 0.018066 | 0.210097 | 8.48E-06 |
| cg02285386 | 0.193288 | 0.093399 | 0.400008 | 9.46E-06 |
| cg02290550 | 0.061526 | 0.01995 | 0.189745 | 1.22E-06 |
| cg02335376 | 0.024777 | 0.00653 | 0.094019 | 5.49E-08 |
| cg02374486 | 14.32767 | 4.248799 | 48.31537 | 1.77E-05 |
| cg02576938 | 0.134718 | 0.054472 | 0.333179 | 1.43E-05 |
| cg02608453 | 0.23244 | 0.120414 | 0.448687 | 1.37E-05 |
| cg02717339 | 0.163091 | 0.07181 | 0.370403 | 1.47E-05 |
| cg02736280 | 0.109373 | 0.048852 | 0.244875 | 7.39E-08 |
| cg02737321 | 0.00342 | 0.000298 | 0.039228 | 5.08E-06 |
| cg02774856 | 3.58E-05 | 4.31E-07 | 0.002978 | 5.65E-06 |
| cg02851047 | 0.083644 | 0.027393 | 0.255406 | 1.32E-05 |
| cg02865595 | 0.109793 | 0.041931 | 0.287487 | 6.85E-06 |
| cg03000846 | 0.127945 | 0.052164 | 0.313813 | 7.06E-06 |
| cg03154077 | 47.78862 | 9.02586 | 253.0233 | 5.44E-06 |
| cg03241502 | 0.001239 | 5.89E-05 | 0.026066 | 1.66E-05 |
| cg03331514 | 0.106942 | 0.038383 | 0.297962 | 1.90E-05 |
| cg03339910 | 0.083512 | 0.028847 | 0.241767 | 4.70E-06 |
| cg03520342 | 4.552539 | 2.275378 | 9.108646 | 1.84E-05 |
| cg03750567 | 0.097419 | 0.036574 | 0.259487 | 3.18E-06 |
| cg03835987 | 0.094204 | 0.036629 | 0.24228 | 9.52E-07 |
| cg03880841 | 0.00117 | 5.37E-05 | 0.025483 | 1.75E-05 |
| cg03950476 | 0.074543 | 0.026473 | 0.209903 | 8.86E-07 |
| cg04020309 | 0.164667 | 0.077368 | 0.350473 | 2.86E-06 |
| cg04177132 | 0.01449 | 0.002157 | 0.097322 | 1.32E-05 |
| cg04247135 | 0.083841 | 0.028419 | 0.247342 | 7.09E-06 |
| cg04357965 | 0.110664 | 0.042116 | 0.290781 | 7.97E-06 |
| cg04638014 | 0.210601 | 0.104756 | 0.423389 | 1.23E-05 |
| cg04650656 | 0.191964 | 0.08992 | 0.409808 | 2.00E-05 |
| cg04661436 | 0.129559 | 0.06056 | 0.277171 | 1.39E-07 |
| cg04803153 | 0.128241 | 0.054056 | 0.304238 | 3.17E-06 |
| cg04833514 | 0.081184 | 0.028987 | 0.227371 | 1.76E-06 |
| cg04966159 | 0.127946 | 0.052257 | 0.313265 | 6.78E-06 |
| cg04993257 | 0.05005 | 0.013766 | 0.181968 | 5.44E-06 |
| cg05044414 | 0.100638 | 0.036406 | 0.278198 | 9.59E-06 |
| cg05064489 | 0.141447 | 0.058485 | 0.342089 | 1.42E-05 |
| cg05087067 | 0.105044 | 0.045326 | 0.243442 | 1.48E-07 |
| cg05210373 | 0.125223 | 0.051695 | 0.303331 | 4.17E-06 |
| cg05361406 | 0.032824 | 0.007367 | 0.146259 | 7.41E-06 |
| cg05666287 | 0.141939 | 0.059714 | 0.337388 | 9.89E-06 |
| cg05708497 | 0.035494 | 0.010262 | 0.122768 | 1.34E-07 |
| cg05937445 | 0.078129 | 0.025957 | 0.235162 | 5.77E-06 |
| cg05949660 | 0.058853 | 0.020143 | 0.171956 | 2.24E-07 |
| cg06311355 | 0.045971 | 0.012988 | 0.162718 | 1.79E-06 |
| cg06414073 | 0.011451 | 0.001506 | 0.087089 | 1.57E-05 |
| cg06707910 | 0.029537 | 0.006001 | 0.145387 | 1.48E-05 |
| cg06759518 | 0.049228 | 0.014034 | 0.172677 | 2.56E-06 |
| cg06885782 | 0.088445 | 0.033491 | 0.233568 | 9.82E-07 |
| cg07005444 | 0.023499 | 0.006165 | 0.089568 | 3.93E-08 |
| cg07156249 | 4.098032 | 2.221697 | 7.559029 | 6.32E-06 |
| cg07343703 | 0.166476 | 0.079133 | 0.350224 | 2.30E-06 |
| cg07497569 | 0.089274 | 0.030935 | 0.257638 | 7.90E-06 |
| cg07621169 | 0.164235 | 0.075711 | 0.356265 | 4.83E-06 |
| cg07664173 | 0.000486 | 1.72E-05 | 0.013706 | 7.53E-06 |
| cg07695566 | 0.143713 | 0.06062 | 0.340704 | 1.06E-05 |
| cg07808761 | 0.076736 | 0.028962 | 0.203314 | 2.41E-07 |
| cg07839457 | 4.536508 | 2.313126 | 8.89701 | 1.08E-05 |
| cg07871024 | 0.094783 | 0.038174 | 0.235335 | 3.82E-07 |
| cg08100069 | 0.077545 | 0.027993 | 0.214812 | 8.72E-07 |
| cg08100159 | 0.186361 | 0.097018 | 0.357976 | 4.55E-07 |
| cg08157684 | 0.005112 | 0.000575 | 0.045493 | 2.24E-06 |
| cg08310837 | 0.07776 | 0.02462 | 0.245597 | 1.34E-05 |
| cg08345719 | 0.07893 | 0.026771 | 0.232711 | 4.17E-06 |
| cg08397758 | 0.067222 | 0.020837 | 0.216861 | 6.25E-06 |
| cg08402572 | 0.18858 | 0.089274 | 0.398351 | 1.23E-05 |
| cg08469215 | 0.126652 | 0.049835 | 0.321874 | 1.41E-05 |
| cg08583411 | 0.050056 | 0.014621 | 0.171367 | 1.85E-06 |
| cg08594606 | 0.153439 | 0.074382 | 0.316521 | 3.90E-07 |
| cg08877374 | 0.067757 | 0.020356 | 0.22553 | 1.15E-05 |
| cg09214398 | 0.213666 | 0.113466 | 0.402352 | 1.76E-06 |
| cg09233429 | 0.10129 | 0.040248 | 0.254915 | 1.16E-06 |
| cg09468328 | 0.016127 | 0.003443 | 0.075542 | 1.62E-07 |
| cg09514174 | 0.041649 | 0.010627 | 0.16323 | 5.09E-06 |
| cg09730719 | 0.027603 | 0.006627 | 0.114968 | 8.16E-07 |
| cg10114555 | 0.027602 | 0.005697 | 0.133723 | 8.23E-06 |
| cg10277175 | 0.114003 | 0.044519 | 0.291939 | 6.00E-06 |
| cg10369594 | 0.045922 | 0.014588 | 0.144561 | 1.40E-07 |
| cg10536276 | 0.083622 | 0.028076 | 0.249063 | 8.34E-06 |
| cg10615591 | 0.156246 | 0.067519 | 0.361567 | 1.45E-05 |
| cg10643916 | 0.035563 | 0.008397 | 0.150619 | 5.89E-06 |
| cg10661163 | 0.05732 | 0.015413 | 0.21317 | 1.99E-05 |
| cg10950111 | 0.212196 | 0.104486 | 0.430943 | 1.80E-05 |
| cg10961323 | 0.219806 | 0.11048 | 0.437318 | 1.59E-05 |
| cg10975001 | 0.050656 | 0.014245 | 0.180132 | 4.06E-06 |
| cg10999598 | 0.049346 | 0.012614 | 0.193039 | 1.54E-05 |
| cg11058730 | 3.8E+08 | 60226.7 | 2.4E+12 | 9.65E-06 |
| cg11063729 | 0.046778 | 0.012561 | 0.174204 | 5.00E-06 |
| cg11103390 | 0.090779 | 0.032479 | 0.253728 | 4.76E-06 |
| cg11187245 | 7.567096 | 3.269303 | 17.51473 | 2.28E-06 |
| cg11274940 | 0.033466 | 0.009399 | 0.119151 | 1.58E-07 |
| cg11523350 | 0.169675 | 0.080601 | 0.357185 | 3.00E-06 |
| cg11613875 | 356548.5 | 1638.849 | 77570803 | 3.24E-06 |
| cg11817892 | 0.193861 | 0.09527 | 0.394479 | 6.00E-06 |
| cg11863058 | 0.139199 | 0.06616 | 0.292872 | 2.04E-07 |
| cg12041075 | 0.068482 | 0.023642 | 0.198366 | 7.77E-07 |
| cg12103475 | 0.016796 | 0.002746 | 0.102726 | 9.74E-06 |
| cg12135769 | 0.121612 | 0.049605 | 0.298147 | 4.13E-06 |
| cg12433575 | 0.182356 | 0.085166 | 0.390458 | 1.18E-05 |
| cg12504148 | 0.114942 | 0.045783 | 0.288571 | 4.10E-06 |
| cg12513686 | 0.170487 | 0.07951 | 0.365561 | 5.47E-06 |
| cg12532266 | 0.122284 | 0.053335 | 0.280366 | 6.91E-07 |
| cg12537329 | 0.146663 | 0.065517 | 0.328312 | 3.03E-06 |
| cg12748890 | 0.125568 | 0.052713 | 0.299116 | 2.80E-06 |
| cg12973017 | 0.044837 | 0.010931 | 0.183915 | 1.62E-05 |
| cg13095627 | 0.172797 | 0.093386 | 0.319736 | 2.25E-08 |
| cg13150534 | 0.096694 | 0.034389 | 0.271886 | 9.47E-06 |
| cg13206063 | 0.062929 | 0.025237 | 0.156914 | 2.98E-09 |
| cg13296093 | 0.049222 | 0.013389 | 0.180951 | 5.80E-06 |
| cg13646917 | 0.026874 | 0.006071 | 0.118971 | 1.89E-06 |
| cg13761843 | 0.036395 | 0.008861 | 0.149495 | 4.30E-06 |
| cg13772815 | 0.081052 | 0.028384 | 0.231442 | 2.68E-06 |
| cg13857119 | 0.084692 | 0.028335 | 0.253144 | 9.91E-06 |
| cg14019050 | 0.076532 | 0.029881 | 0.196011 | 8.50E-08 |
| cg14080585 | 0.082358 | 0.029672 | 0.228592 | 1.64E-06 |
| cg14091103 | 0.099758 | 0.034727 | 0.286567 | 1.86E-05 |
| cg14131038 | 0.072059 | 0.021586 | 0.240551 | 1.90E-05 |
| cg14173258 | 0.104751 | 0.038883 | 0.2822 | 8.12E-06 |
| cg14204433 | 0.066874 | 0.020702 | 0.216027 | 6.15E-06 |
| cg14218480 | 0.082287 | 0.030258 | 0.223778 | 9.94E-07 |
| cg14292823 | 0.0539 | 0.016368 | 0.177489 | 1.56E-06 |
| cg14333539 | 0.087996 | 0.034566 | 0.224016 | 3.43E-07 |
| cg14353649 | 0.043759 | 0.011037 | 0.173497 | 8.50E-06 |
| cg14413165 | 0.010771 | 0.002105 | 0.055123 | 5.36E-08 |
| cg14550760 | 0.079552 | 0.025714 | 0.246115 | 1.12E-05 |
| cg14591340 | 0.146134 | 0.061748 | 0.345841 | 1.21E-05 |
| cg14809332 | 0.038117 | 0.009645 | 0.150643 | 3.17E-06 |
| cg14896463 | 0.027543 | 0.005311 | 0.14285 | 1.89E-05 |
| cg15128365 | 0.092542 | 0.036077 | 0.237382 | 7.34E-07 |
| cg15287850 | 0.167207 | 0.075414 | 0.370729 | 1.07E-05 |
| cg15299835 | 0.022408 | 0.004123 | 0.121787 | 1.09E-05 |
| cg15379633 | 0.063236 | 0.019023 | 0.210207 | 6.64E-06 |
| cg15409237 | 0.138362 | 0.060543 | 0.316208 | 2.73E-06 |
| cg15442268 | 0.013759 | 0.002433 | 0.077811 | 1.24E-06 |
| cg15537850 | 0.062822 | 0.019441 | 0.203003 | 3.76E-06 |
| cg15825417 | 0.059136 | 0.01728 | 0.202379 | 6.63E-06 |
| cg16019612 | 0.095031 | 0.032976 | 0.273867 | 1.31E-05 |
| cg16121744 | 0.092548 | 0.033362 | 0.256732 | 4.83E-06 |
| cg16278747 | 0.091047 | 0.033461 | 0.247737 | 2.70E-06 |
| cg16393207 | 0.129114 | 0.060861 | 0.273913 | 9.58E-08 |
| cg16449084 | 0.075916 | 0.027818 | 0.207179 | 4.83E-07 |
| cg16495212 | 0.099036 | 0.034929 | 0.280805 | 1.37E-05 |
| cg16622899 | 0.04884 | 0.013095 | 0.182151 | 6.94E-06 |
| cg16859636 | 0.199017 | 0.096983 | 0.408398 | 1.07E-05 |
| cg17001035 | 0.032038 | 0.007115 | 0.144271 | 7.40E-06 |
| cg17079034 | 0.019719 | 0.003617 | 0.107491 | 5.69E-06 |
| cg17186163 | 0.012284 | 0.00218 | 0.069209 | 6.11E-07 |
| cg17209284 | 0.13906 | 0.064695 | 0.298905 | 4.35E-07 |
| cg17317391 | 0.126112 | 0.054399 | 0.29236 | 1.39E-06 |
| cg17327990 | 1.96E-06 | 5.43E-09 | 0.00071 | 1.23E-05 |
| cg17330838 | 0.09312 | 0.035441 | 0.244669 | 1.46E-06 |
| cg17393635 | 0.097219 | 0.034717 | 0.272249 | 9.15E-06 |
| cg17397004 | 0.12713 | 0.049704 | 0.325167 | 1.67E-05 |
| cg17453840 | 0.178925 | 0.08693 | 0.368275 | 2.98E-06 |
| cg17656260 | 0.000239 | 5.81E-06 | 0.009873 | 1.11E-05 |
| cg17720013 | 0.123009 | 0.057896 | 0.261354 | 5.04E-08 |
| cg17792192 | 0.328872 | 0.200781 | 0.538682 | 1.00E-05 |
| cg18030218 | 2.02E+34 | 1.61E+21 | 2.54E+47 | 2.85E-07 |
| cg18183774 | 0.169577 | 0.080211 | 0.358509 | 3.39E-06 |
| cg18359371 | 0.026143 | 0.006155 | 0.11104 | 7.88E-07 |
| cg18489607 | 0.037458 | 0.011166 | 0.125659 | 1.04E-07 |
| cg18511546 | 26.13639 | 7.589013 | 90.01316 | 2.31E-07 |
| cg18649823 | 0.000237 | 5.54E-06 | 0.010168 | 1.34E-05 |
| cg18729787 | 0.08958 | 0.032162 | 0.249508 | 3.91E-06 |
| cg18869127 | 0.062357 | 0.019418 | 0.200243 | 3.14E-06 |
| cg18924331 | 0.150254 | 0.065096 | 0.346817 | 8.94E-06 |
| cg19028462 | 0.140337 | 0.066056 | 0.298148 | 3.26E-07 |
| cg19300923 | 0.110882 | 0.042188 | 0.291433 | 8.17E-06 |
| cg19482025 | 0.072448 | 0.02411 | 0.217699 | 2.93E-06 |
| cg19577312 | 0.07233 | 0.02403 | 0.217708 | 2.99E-06 |
| cg19619576 | 0.187995 | 0.089715 | 0.393936 | 9.51E-06 |
| cg19729479 | 92.21792 | 13.17397 | 645.5263 | 5.19E-06 |
| cg19878482 | 0.108576 | 0.041192 | 0.286192 | 7.12E-06 |
| cg19887750 | 0.02342 | 0.004643 | 0.118133 | 5.44E-06 |
| cg20091689 | 0.176193 | 0.084466 | 0.367532 | 3.69E-06 |
| cg20321801 | 0.052132 | 0.014613 | 0.185983 | 5.31E-06 |
| cg20655350 | 0.092697 | 0.032652 | 0.263167 | 7.91E-06 |
| cg20801110 | 0.151019 | 0.065535 | 0.348006 | 9.07E-06 |
| cg20831708 | 0.166562 | 0.078842 | 0.35188 | 2.64E-06 |
| cg21161394 | 0.070109 | 0.022784 | 0.215733 | 3.58E-06 |
| cg21163717 | 14.18607 | 4.26298 | 47.2075 | 1.53E-05 |
| cg21184369 | 0.245054 | 0.128817 | 0.466175 | 1.82E-05 |
| cg21548131 | 0.137742 | 0.064759 | 0.292978 | 2.63E-07 |
| cg21655969 | 0.00254 | 0.000182 | 0.035401 | 8.76E-06 |
| cg21800196 | 0.112717 | 0.048285 | 0.263129 | 4.50E-07 |
| cg21824343 | 0.094876 | 0.040568 | 0.221886 | 5.53E-08 |
| cg22027399 | 0.049377 | 0.013781 | 0.176915 | 3.84E-06 |
| cg22128918 | 0.023429 | 0.005542 | 0.099042 | 3.33E-07 |
| cg22271905 | 0.013678 | 0.002136 | 0.087577 | 5.88E-06 |
| cg22306579 | 0.037217 | 0.009013 | 0.153671 | 5.40E-06 |
| cg22493877 | 0.019842 | 0.004173 | 0.09434 | 8.32E-07 |
| cg22595230 | 0.140397 | 0.057107 | 0.345165 | 1.89E-05 |
| cg22704780 | 0.073227 | 0.024839 | 0.215878 | 2.15E-06 |
| cg22923006 | 0.188978 | 0.08822 | 0.404813 | 1.81E-05 |
| cg23075364 | 0.112902 | 0.043861 | 0.290622 | 6.14E-06 |
| cg23120601 | 0.129792 | 0.054687 | 0.308046 | 3.65E-06 |
| cg23152216 | 0.06089 | 0.023266 | 0.159356 | 1.19E-08 |
| cg23244095 | 0.089893 | 0.031457 | 0.256888 | 6.90E-06 |
| cg23285459 | 0.277366 | 0.157765 | 0.487638 | 8.40E-06 |
| cg23288103 | 0.073305 | 0.02494 | 0.215467 | 2.03E-06 |
| cg23359895 | 779560.7 | 2182.651 | 2.78E+08 | 6.08E-06 |
| cg23696248 | 0.130586 | 0.052906 | 0.322324 | 1.01E-05 |
| cg23725321 | 0.159914 | 0.071837 | 0.355979 | 7.13E-06 |
| cg23902550 | 0.055854 | 0.01588 | 0.19645 | 6.92E-06 |
| cg23923934 | 9.023151 | 3.552367 | 22.91916 | 3.74E-06 |
| cg23925513 | 0.050828 | 0.015143 | 0.170605 | 1.42E-06 |
| cg23964386 | 0.019854 | 0.00376 | 0.104833 | 3.90E-06 |
| cg24179027 | 0.052309 | 0.018171 | 0.150581 | 4.51E-08 |
| cg24367850 | 0.061398 | 0.017069 | 0.220849 | 1.93E-05 |
| cg24404823 | 0.224282 | 0.116825 | 0.430577 | 7.05E-06 |
| cg24408057 | 0.081207 | 0.027258 | 0.241928 | 6.55E-06 |
| cg24766690 | 0.01721 | 0.002789 | 0.106209 | 1.21E-05 |
| cg24768595 | 0.220632 | 0.113633 | 0.428384 | 8.04E-06 |
| cg24915511 | 0.004139 | 0.000418 | 0.04101 | 2.74E-06 |
| cg25004071 | 0.139889 | 0.05868 | 0.333487 | 9.10E-06 |
| cg25141995 | 0.10785 | 0.043512 | 0.267323 | 1.52E-06 |
| cg25221637 | 0.098987 | 0.036727 | 0.266791 | 4.83E-06 |
| cg25446191 | 0.089572 | 0.030123 | 0.266346 | 1.43E-05 |
| cg25591888 | 0.142782 | 0.058985 | 0.345621 | 1.59E-05 |
| cg25635864 | 0.02134 | 0.004071 | 0.11187 | 5.33E-06 |
| cg25900902 | 0.156865 | 0.072396 | 0.339889 | 2.66E-06 |
| cg26099902 | 0.163769 | 0.074411 | 0.360435 | 6.95E-06 |
| cg26136365 | 0.033008 | 0.006989 | 0.155899 | 1.66E-05 |
| cg26299084 | 0.062049 | 0.017577 | 0.21904 | 1.56E-05 |
| cg26418434 | 0.096279 | 0.03686 | 0.25148 | 1.77E-06 |
| cg26617637 | 0.1155 | 0.04593 | 0.290444 | 4.48E-06 |
| cg26672452 | 0.067987 | 0.025304 | 0.182665 | 9.74E-08 |
| cg26741686 | 0.189059 | 0.094507 | 0.378209 | 2.50E-06 |
| cg26897283 | 0.034895 | 0.008247 | 0.147656 | 5.14E-06 |
| cg27048142 | 0.120305 | 0.056862 | 0.25453 | 3.05E-08 |
| cg27285720 | 4.141154 | 2.33431 | 7.346564 | 1.18E-06 |
| cg27313492 | 0.067619 | 0.020163 | 0.226767 | 1.28E-05 |
| cg27518898 | 0.064043 | 0.020063 | 0.20443 | 3.47E-06 |
| cg27529346 | 9.460346 | 3.369121 | 26.56424 | 1.99E-05 |
