# Supplementary material for: Identification of Melanoma Subsets Based on DNA Methylation Sites and Construction of a Prognosis Evaluation Model
Source: J Oncol. 2022 Oct 11;2022:6608650. doi: 10.1155/2022/6608650 (PMC9578801; doi:10.1155/2022/6608650)
Supplement: Supplementary Materials — Supplementary Table 1. Prognosis-related methylation sites by univariate Cox regression analysis (783 sites were found, P < 0.0001). Supplementary Table 2. Multivariate Cox regression analysis of the 783 methylation sites (256 sites were found, P < 0.0001). Supplementary Table 3. Level of the 256 sites in 338 samples and the follow-up. Supplementary Table 4. Analysis of differences in methylation site levels between the 7 clusters. Supplementary Table 5. Risk assessment. Supplementary Table 6. Testing the prediction model in 60% of the samples (randomly) for 100 times. Supplementary Table 7. Functional enrichment analysis of genes and 28 pathways were observed. Supplementary Table 8. The correlation coefficients between the expression levels of the 35 hub genes (∗∗P < 0.01, ∗P < 0.05). Supplementary Table 9. The correlation coefficients of the critical genes validated in the testing group (∗∗P < 0.01). Figure S1. The clinical features in different DNA methylation subgroups. The clinical T categories (a), N stage (b), and M status (c) of different subtypes. The tumor stage (d), patient ages (e), and gender (f) in different subtypes. C, cluster; T, primary tumor; N, lymph node involvement; M, distant metastases. [file 6608650.f1.zip › Supplementary Table 7. Functional enrichment analysis of genes and 28 pathways were observed.docx]

| **Supplementary Table 7. Functional enrichment analysis of genes and 28 pathways were observed.** | | | |
| --- | --- | --- | --- |
| **Pathway** | **Counts** | ***P*-value** | **Genes** |
| Human cytomegalovirus infection | 12 | 1.020╳10^-6^ | AKT3, GNA12, HLA-B, HLA-C, IL10RB, KRAS, PLCB2, PTK2, RAC3, TAP1, TP53, TSC2 |
| Herpes simplex virus 1 infection | 8 | 0.000287 | EEF1D, HLA-B, HLA-C, HLA-DMA, PER3, TAF4, TAP1, TP53 |
| Cellular senescence | 7 | 0.000643 | AKT3, HLA-B, HLA-C, KRAS, TP53, TSC2, VDAC1 |
| Phospholipase D signaling pathway | 6 | 0.0023 | AKT3, CYTH1, GNA12, KRAS, PLCB2, TSC2 |
| Natural killer cell mediated cytotoxicity | 6 | 0.00134 | CD247, HLA-B, HLA-C, KRAS, PRF1, RAC3 |
| Sphingolipid signaling pathway | 6 | 0.000751 | AKT3, GNA12, KRAS, PLCB2, RAC3, TP53 |
| Viral myocarditis | 6 | 1.520╳10^-5^ | EIF4G1, HLA-B, HLA-C, HLA-DMA, PRF1, RAC3 |
| Thyroid hormone signaling pathway | 5 | 0.00409 | AKT3, KRAS, PLCB2, TP53, TSC2 |
| Progesterone-mediated oocyte maturation | 4 | 0.0125 | AKT3, CDC25B, CPEB1, KRAS |
| Choline metabolism in cancer | 4 | 0.0125 | AKT3, KRAS, RAC3, TSC2 |
| Longevity regulating pathway | 4 | 0.0087 | AKT3, KRAS, TP53, TSC2 |
| Colorectal cancer | 4 | 0.00773 | AKT3, KRAS, RAC3, TP53 |
| ErbB signaling pathway | 4 | 0.00742 | AKT3, KRAS, NCK2, PTK2 |
| Antigen processing and presentation | 4 | 0.00524 | HLA-B, HLA-C, HLA-DMA, TAP1 |
| Pancreatic cancer | 4 | 0.00477 | AKT3, KRAS, RAC3, TP53 |
| B cell receptor signaling pathway | 4 | 0.00392 | AKT3, CD81, KRAS, RAC3 |
| VEGF signaling pathway | 4 | 0.002 | AKT3, KRAS, PTK2, RAC3 |
| Autoimmune thyroid disease | 4 | 0.00124 | HLA-B, HLA-C, HLA-DMA, PRF1 |
| Type I diabetes mellitus | 4 | 0.000606 | HLA-B, HLA-C, HLA-DMA, PRF1 |
| Graft-versus-host disease | 4 | 0.000505 | HLA-B, HLA-C, HLA-DMA, PRF1 |
| Allograft rejection | 4 | 0.000376 | HLA-B, HLA-C, HLA-DMA, PRF1 |
| Glioma | 3 | 0.0306 | AKT3, KRAS, TP53 |
| Melanoma | 3 | 0.0275 | AKT3, KRAS, TP53 |
| Fc epsilon RI signaling pathway | 3 | 0.0237 | AKT3, KRAS, RAC3 |
| Non-small cell lung cancer | 3 | 0.022 | AKT3, KRAS, TP53 |
| Central carbon metabolism in cancer | 3 | 0.0211 | AKT3, KRAS, TP53 |
| Long-term depression | 3 | 0.0171 | GNA12, KRAS, PLCB2 |
| Endometrial cancer | 3 | 0.0156 | AKT3, KRAS, TP53 |
